# A Two-Dimensional Grid-Cell Code for Three-Dimensional Navigation in Freely Flying Bats

**DOI:** 10.64898/2026.06.10.728358

**Published:** 2026-06-11

**Authors:** Kevin K. Qi, Michael M. Yartsev

**Affiliations:** 1 Biophysics Graduate Group, UC Berkeley, Berkeley, California 94720, United States.; 2 Department of Bioengineering, UC Berkeley, Berkeley, California 94720, United States.; 3 Department of Neuroscience, UC Berkeley, Berkeley, California 94720, United States.; 4 Helen Wills Neuroscience Institute, UC Berkeley, Berkeley, California 94720, United States.; 5 Howard Hughes Medical Institute, University of California, Berkeley, Berkeley, CA, USA

## Abstract

Animals have evolved to navigate environments with diverse geometries and dimensionalities, yet how neural circuits for spatial navigation support behavior across such varied demands remains unresolved. In two-dimensional environments, grid cells in the medial entorhinal cortex provide a periodic signal for self-location that is thought to arise from underlying toroidal attractor dynamics at the ensemble level. However, during movement in three-dimensional environments, studies have reported a loss of global grid-cell periodicity. Furthermore, it remains unknown whether, at the ensemble level, a toroidal attractor is preserved across species and, if so, how such a two-dimensional code could support navigation in three-dimensional space. Here we performed large-scale, wireless neural recordings from the medial entorhinal cortex of freely flying bats engaged in spontaneous aerial foraging. We find that grid cells exhibit robust periodic firing during structured flight trajectories, and that co-modular grid-cell ensembles show topological signatures consistent with a two-dimensional toroidal manifold. Behavioral analyses of bats navigating in the wild and in the laboratory revealed that individual flight paths are organized along two-dimensional planes of motion, providing a natural substrate for a two-dimensional code. Accordingly, toroidal phase along flights was consistent with plane-wave patterns on the plane of motion, and single-cell firing was well described by a hexagonal lattice on the same plane. Together, these findings reveal a parsimonious solution whereby a two-dimensional neural code aligns with behaviorally relevant subspaces to support navigation in a three-dimensional world.

## Introduction

Spatial navigation relies on internal representations of position^1,2^. In mammals, a key component of this system are grid cells in the medial entorhinal cortex, whose firing fields form a periodic lattice in two-dimensional (2D) environments^1,3–5^. In rodents, this spatial periodicity is thought to arise from population dynamics evolving on a low-dimensional toroidal manifold, as formalized in continuous attractor network (CAN) models^1,6–16^. Yet, whether and how such computations extend to three-dimensional environments remains unresolved. Although many animals live and move in three-dimensional (3D) space, prior studies have suggested that grid-cell periodicity becomes disrupted during 3D movement^17,18^, and it remains unknown whether the toroidal network dynamics reported in rodents generalize to other species, particularly those that specialize in three-dimensional navigation. Furthermore, even if toroidal grid-cell organization is conserved across species, it remains unclear how a fundamentally two-dimensional neural code can support navigation in a three-dimensional environment. This dimensionality mismatch creates a fundamental representational challenge: a two-dimensional toroidal manifold provides a natural neural substrate for 2D positional coding, yet it can only represent one of many possible two-dimensional subspaces within 3D space.

Here we address these gaps by wirelessly recording large-scale ensemble activity from the medial entorhinal cortex of freely flying bats (*Rousettus aegyptiacus*) during spontaneous, unconstrained three-dimensional spatial behavior. This approach allowed us to directly test (i) whether periodic firing of grid cells is preserved during three-dimensional movement, (ii) whether the underlying population dynamics are consistent with a toroidal organization, and (iii) how a two-dimensional neural code could support navigation in a three-dimensional world.

## Results

### Periodic grid-cells in flying bats

**Figure 1.**
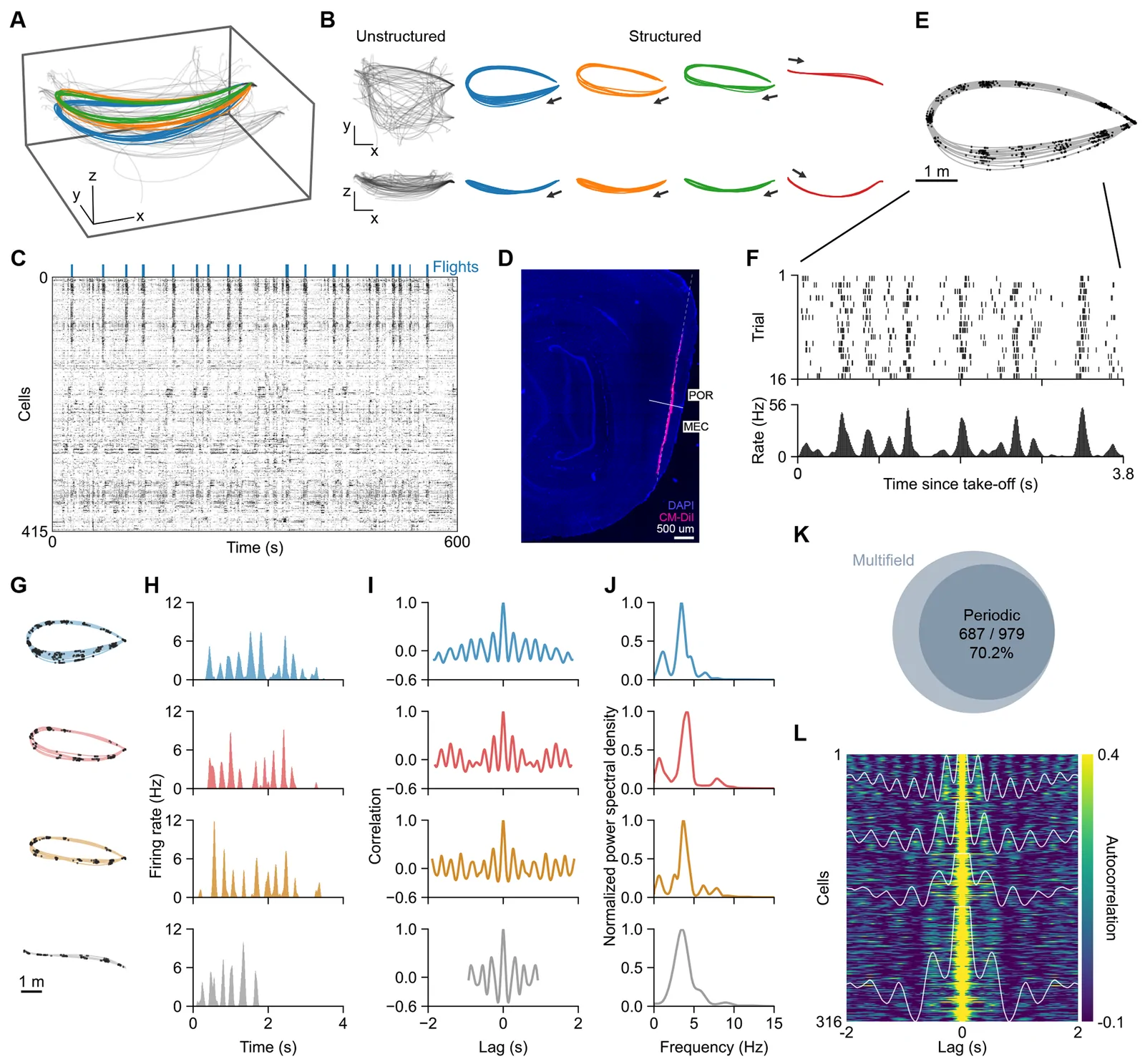
Periodic grid-cells in flying bats **(A)** Structured (colored) and unstructured (grey) flight trajectories from a representative recording session in the flight room. Scale bar, 1 m. **(B)** Top and side views (top and bottom rows, respectively) of structured (colored) and unstructured (grey) flight paths from the same session as in **A**. Arrows indicate flight start direction. Scale bar, 1 m. **(C)** Rastermap^47^ visualization of the neural activity of 415 simultaneously recorded cells during a 600-s segment of a recording session. Blue bars indicate flights. **(D)** Sagittal section from one recorded bat showing Neuropixels 1.0 probe track through the medial entorhinal cortex (MEC; white dashed line), stained for 4′,6-diamidino-2-phenylindole (DAPI, blue) and CM-DiI (pink) (Methods). POR, postrhinal cortex. Data were collected from the probe contacts located in MEC. **(E)** Spikes from an example MEC neuron (black) overlaid on flight trajectories (grey). **(F)**, Top, raster plot of the neuron in **E** aligned to flight take-off. Bottom, trial-averaged firing rate aligned to take-off. **(G)** Spikes of an example grid-cell (black dots) across four different flight trajectories (colored). Note the conserved periodic firing across the different flight trajectories. **(H)** Trial averaged firing rate of the grid-cell in **G** aligned to take-off. **(I)** Autocorrelation of the trial average firing rate in **H**. **(J)** Peak normalized power spectral density of the firing rate modulation shown in **H**, revealing conserved periodic structure. **(K)** Percentage of multifield cells classified as significantly periodic (Methods). **(L)** Time-lagged firing rate autocorrelograms of significantly periodic grid cells, clustered across sessions by their power spectral profile and sorted by mean cluster grid spacing. White traces indicate mean autocorrelation of each cluster. Only clusters with 50 cells or more are shown.

To examine grid cells during 3D navigation, we used Neuropixels to wirelessly record single-unit activity from the superficial layers II/III of the medial entorhinal cortex (MEC) of freely flying bats engaged in spontaneous aerial foraging^19–21^ (n = 3 bats, 13 sessions; Figures 1A–1D; Methods). Bats naturally developed a diverse repertoire of self-selected flight paths, many of which were highly structured and repeated at high precision^20^. Most of the bat’s flight trajectories could be unambiguously grouped into distinct clusters, with the remainder classified as unstructured flights (Figure 1B; 68.4% or 662 of 968 flights, structured; 31.6% or 306 of 968 flights, unstructured across all bats). We recorded a total of 2,353 well isolated single units (181 ± 111 mean ± s.d. per session), of which 979 exhibited multiple and stable firing fields along structured flights (Figures 1E, 1F, and S1; Methods). Importantly, many of these units exhibited highly periodic firing patterns that extended throughout the flight. This global periodicity was maintained both within (Figures 1E and 1F) and across flight trajectories (Figure 1G). Leveraging the natural trial structure that emerged spontaneously during foraging, we quantified the firing periodicity using the autocorrelation and power spectral densities (PSD) of trial-averaged linearized firing rates (Figures 1H–1J, Methods). More than 70% of the multifield units exhibited robust periodic firing along structured flight paths (687/979 or 70.2%; Figure 1K). Moreover, clustering of PSD profiles revealed groupings of units with similar grid spacing (Figure 1L), arranged with increasing scale from the dorsal to ventral axis of the MEC (Figure S2), reminiscent of the modular organization of grid cells in rodents^22^. Combined, these findings reveal that global grid-cell periodicity is robustly preserved during spontaneous movement in a three-dimensional space.

### Toroidal topology of grid-cell population activity in flying bats

**Figure 2.**
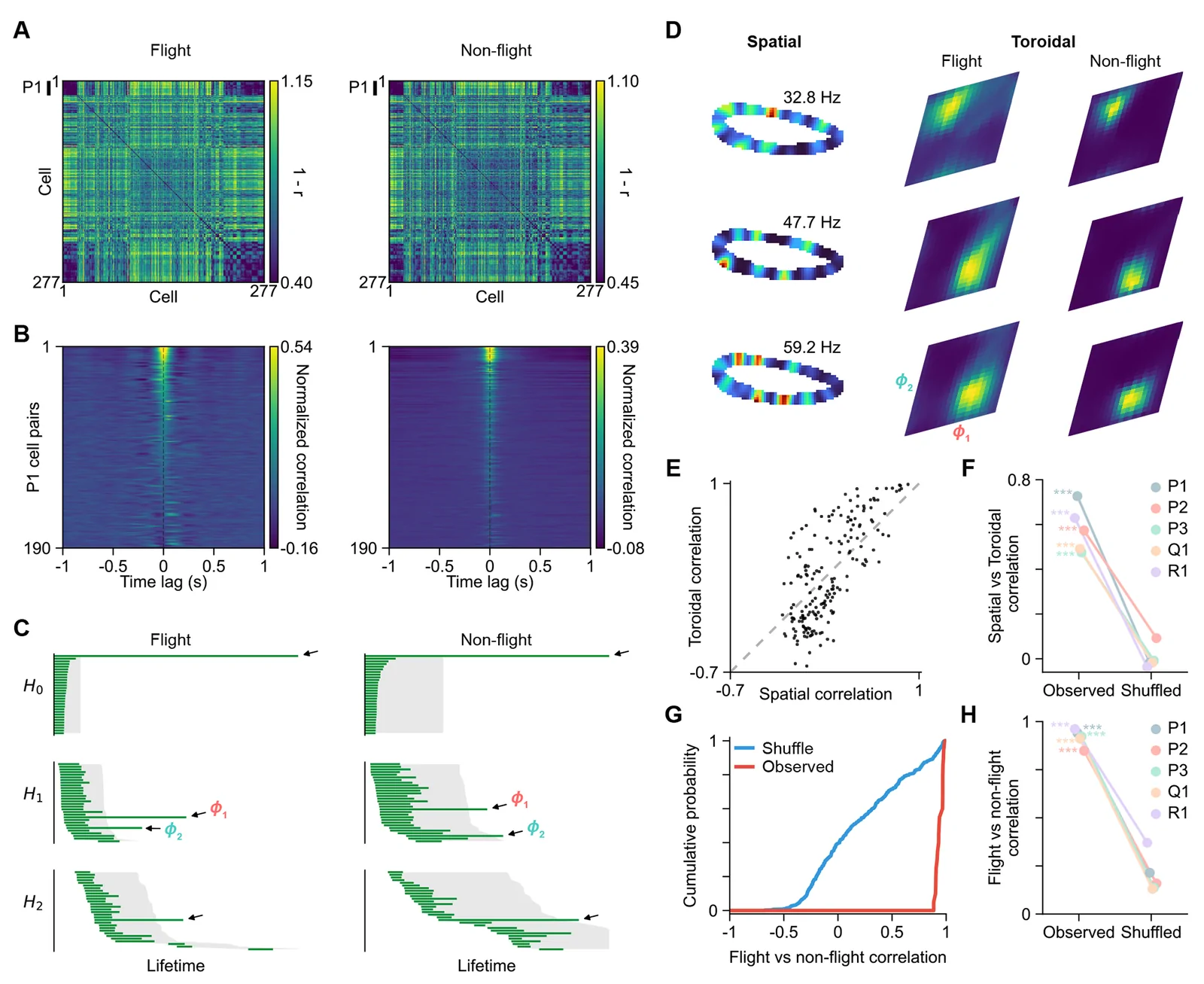
Toroidal topology of grid-cell population activity in flying bats **(A)** Clustered and sorted pairwise correlation distance matrix between simultaneously recorded cells during flight (left) and non-flight (right) periods (Methods). Clustering and sorting were computed using flight data only and applied to the non-flight distance matrix. P1 indicates a putative grid module. **(B)** Normalized cross-correlograms for all cell-pairs of the module shown in **A** during flight (left) and non-flight (right), ordered by zero-lag correlation measured during flight. Cross-correlations were normalized by mean-subtraction. **(C)** Persistent cohomology analysis of co-modular P1 population activity during flight (left) and non-flight (right). Barcodes (green) show one long-lived connected component (H0), two one-dimensional cycles (H1), and one two-dimensional cavity (H2), consistent with a two-dimensional torus. Grey shaded area indicates 95th percentile of shuffled lifetimes (Methods). Arrows denote significant topological features of each dimensionality. **(D)** Spatial rate maps and toroidal rate maps of three example grid cells. Spatial rate maps are shown on the left. Peak rate map firing rate is indicated. Toroidal rate maps are shown in the middle for flight bouts and right for non-flight bouts. **(E)** Pairwise spatial correlation (between spatial rate maps) versus pairwise toroidal correlation (between toroidal rate maps during flight) of P1 grid cell pairs (Pearson *r* = 0.73). **(F)** Pearson correlation between pairwise spatial correlation and pairwise toroidal correlation for each of the five grid modules compared to shuffle distribution constructed by random permutation of unit identity. *n* = 190 (P1), 190 (P2), 210 (P3), 378 (Q1), 253 (R1) cell pairs. ***P <0.001. **(G)** Observed and shuffled (red and blue, respectively) cumulative distribution of pairwise correlation between toroidal rate maps during flight and non-flight for the P1 module. Shuffled distribution is constructed by random permutation of unit identity. **(H)** Mean within-module pairwise toroidal rate map correlation during flight and non-flight bouts for each of the five different grid modules versus shuffle as constructed in **F**. *n* = 20 (P1), 20 (P2), 21 (P3), 28 (Q1), 23 (R1) cells. ***P < 0.001.

In rodents, co-modular grid-cell activity exhibits toroidal topology consistent with CAN models^1,6–9,13,14^. We therefore asked whether this organization is also present in the population activity of co-modular grid-cells in bats. Leveraging the stable nature of co-modular grid cell spike-time relations^10–16,23,24^, we identified grid modules using spike-time-correlation-based clustering^13,14^, yielding five modules that exhibited stable within-module spike-time correlations across both flight and non-flight behavioral states (Figures 2A, 2B, and S3). We used persistent cohomology to identify long-lived topological features in the high-dimensional activity of co-modular grid cells, summarized in a barcode of feature lifetimes^13,14,25,26^. Population activity of these modules exhibited prominent features consistent with a two-dimensional torus: a single connected structure, two circular dimensions, and one enclosed cavity, in line with the toroidal topology of grid cell activity observed in rodents^13,14^ (Figures 2C and S4; Methods).

We reasoned that if the spatial activity of grid-cells reflects attractor dynamics on the internal toroidal manifold, then units with similar toroidal tuning should also exhibit more similar spatial firing patterns during flight. To test this, we used cohomological decoding^13,14,25^ to construct a coordinate system on the toroidal manifold, with each circular dimension parameterized by an angular value (Methods). We then computed rate maps of each grid cell with respect to both external Euclidean coordinates (spatial rate maps; Figure 2D, left; Methods) and internal toroidal coordinates (toroidal rate maps; Figure 2D, middle and right, for flight and non-flight, respectively; Methods). Pairwise similarity of co-modular grid cells was strongly correlated between their spatial and toroidal rate maps (Figures 2E and 2F; *r* = 0.58 ± 0.10, mean ± s.d. across modules; all modules: *P* < 0.001; Methods), suggesting that grid cells which exhibit similar spatial firing patterns are also located nearby on the toroidal manifold, as predicted from CAN models and experimental data in rodents^1,6–16,23^. Furthermore, if grid cells operate on an intrinsically defined toroidal manifold, this structure is expected to persist across distinct behavioral states^1,6–16,23^. We therefore asked whether toroidal tuning is preserved between flight and non-flight periods. Indeed, we found that the tuning of grid cells with respect to toroidal coordinates remained stable across behavioral states (Figures 2D, 2G, and 2H; *r* = 0.92 ± 0.04, mean ± s.d. across modules; all modules: *P* < 0.001; see also Figure S5; Methods). Together, these findings demonstrate that co-modular grid cells in flying bats exhibit two-dimensional toroidal population structure, similar to that observed in rodents^13,14^, despite navigation occurring in three-dimensional space.

### Mapping of grid-cell representations on the plane of motion

**Figure 3.**
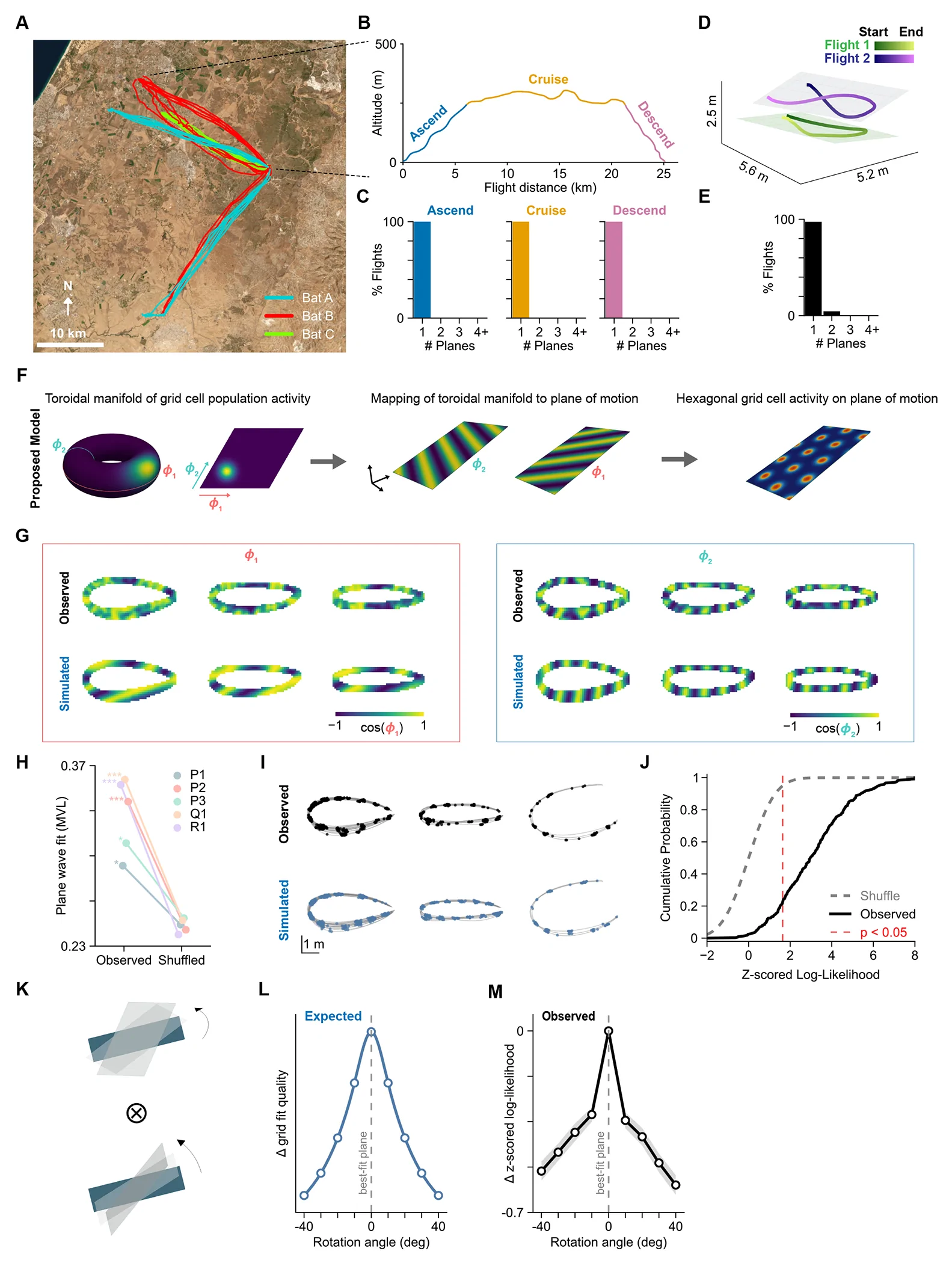
Mapping of grid-cell representations on the plane of motion **(A)** Flight trajectories of three example Egyptian fruit bats (colors) over multiple nights in the wild (individual traces). **(B)** Altitude profile of a representative commute flight, showing ascend (blue), cruise (orange) and descend (pink) phases (Methods). **(C)** Distribution of number of planes required to explain >98% of positional variance during ascent, cruise, and descent phases. Most segments were captured by a single plane. **(D)** Two representative flight paths during spontaneous foraging in laboratory flight room. Color indicates progression from start to end. **(E)** Distribution of number of planes required to explain >98% of the positional variance for laboratory flights (96.4% or 1842/1910 of flights). Most flights were captured by a single plane. **(F)** Proposed model. A two-dimensional toroidal manifold (left, shown as folded and unfolded) is mapped onto the plane of motion of each flight, such that toroidal axes ($\phi_{1}$ and $\phi_{2}$) generate plane-wave components (middle) whose combination gives rise to hexagonal structure on that plane (right). **(G)** Top, observed toroidal phase maps of $\phi_{1}$ (left, red box) and $\phi_{2}$ (right, blue box) on structured flight paths in the plane of motion. Bottom, best-fit plane-waves on the plane of motion. **(H)** Mean vector length (MVL) quantifying the plane-wave fit for each module, compared to shuffled (Methods). Points denote modules (color-coded); lines connect observed and shuffled values. *n* = 20 (P1), 20 (P2), 21 (P3), 28 (Q1), 23 (R1) cells. ***P < 0.001. *P < 0.05. **(I)** Top, observed spikes (black) overlaid on flight trajectories (grey). Bottom, corresponding best-fit simulated spike positions (blue), from an underlying hexagonal lattice on the plane of motion, overlaid on the same flight trajectories. **(J)** Cumulative distribution of z-scored log-likelihood values for hexagonal lattice fits (black) compared with shuffled controls (grey) (Methods). Red dashed line indicates *p* < 0.05 significance threshold. **(K)** Schematic of candidate planes generated by rotating the plane of motion around the azimuth and pitch axes. **(L)** Expected change in grid-fit quality as candidate planes are rotated away from the plane of motion, defined as the best-fit plane to the bat’s flight path. If grid-cell representations are aligned to the plane of motion, grid-fit quality should be maximal at 0° rotation and decrease monotonically with increasing angular deviation. **(M)** Observed mean difference between z-scored log-likelihood on rotated flight planes and best-fit plane of motion. Grey shaded region denotes s.e.m. of the mean trace.

The observed two-dimensional toroidal topology of grid-cell population activity in flying bats raises a fundamental challenge: how can a two-dimensional internal representation support navigation through a three-dimensional environment? This is a non-trivial problem because a toroidal manifold is naturally suited for encoding a two-dimensional space, whereas a three-dimensional volume contains many possible two-dimensional subspaces. To begin addressing this problem we first examined the ethology of bat flight, asking how bats naturally move through three-dimensional environments, both in the wild and in the laboratory. In the wild, Egyptian fruit bats are known to perform nightly commutes extending over dozens of kilometers to remote foraging sites^27–29^. We analyzed GPS tracking data^30^ obtained during such large-scale, outdoor navigation (Figure 3A) and found that bat flight trajectories consistently unfolded as a sequence of three distinct planar segments: (i) an ascent towards a target altitude, (ii) extended flight at a relatively fixed cruising elevation, and finally (iii) a descent towards the foraging site (Figure 3B). Indeed, a single plane was sufficient to account for nearly all the spatial variance in each of the flight segments (Figures 3C and S6A). Thus, large-scale navigation of Egyptian fruit bats in the wild can be described as directional movement unfolding across successive planes of motion.

We next examined the geometry of spontaneous flights during foraging in the laboratory, which occur at spatial scales comparable to natural foraging in the wild (meters rather than kilometers)^27,28^. Remarkably, we observed a similar principle of planar organization: self-selected, spontaneous flight paths were well described by a single 2D plane of motion accounting for the vast majority of spatial variance (Figures 3D, 3E, and S6B). These observations suggest that, despite movement occurring in three-dimensional space, behavior is often structured along lower-dimensional subspaces, providing a natural substrate onto which grid-cell representations can be mapped.

We therefore hypothesized that the dimensional mismatch between a two-dimensional grid code and three-dimensional space could be resolved by mapping grid-cell representations onto the animal’s present plane of motion (Figure 3F). This hypothesis gives direct predictions at both the population and single-cell levels. At the population level, the grid torus should be aligned to the plane of motion, such that its two toroidal axes would correspond to the plane-wave components of a hexagonal lattice on the plane^7,8,13–15^ (Figure 3F, middle; Figures S7A–S7C). At the single-cell level, the firing patterns of individual grid cells along flights should be consistent with an underlying hexagonal lattice defined on the flight plane^15,23^ (Figure 3F, right; Figure S7D).

To begin testing these predictions, we first estimated toroidal phase maps by computing the mean toroidal phase ($\phi_{1}$ and $\phi_{2}$) at each position during flight (Figure 3G; Methods). If the toroidal manifold axes align with the flight plane, the toroidal phase patterns along each flight path should match those resulting from the intersection of the trajectory with plane waves defined on the plane of motion. Consistent with this prediction, fitting plane waves to the toroidal phase maps revealed that the observed phase patterns were well captured by plane waves defined on the flight plane (Figures 3G and 3H; MVL = 0.33 ± 0.03, mean ± s.d. across modules; P2, Q1, R1: *P* < 0.001; P1: *P* = 0.027; P3: *P* = 0.017; Methods). Next, we asked whether the firing patterns of individual grid cells were consistent with a hexagonal lattice on the plane of motion. To do so, we computed the log-likelihood of the observed spike positions under candidate grid patterns spanning the full grid parameter space (Methods). Because this approach operates directly on raw spike positions, it avoids the need for additional hyperparameters for rate map estimation but is sensitive to background and out-of-field spiking activity. We therefore restricted the analysis to a subset of cells with a minimum of two prominent peaks in their interspike distance distribution (n = 332; Methods). We found that over 70% of the cells exhibited firing patterns consistent with a hexagonal lattice on the plane of motion (n = 236/332, 71.1%; Figures 3I, 3J, and S8; Methods).

To further assess the specificity of this mapping, we repeated the analysis on 48 nearby candidate planes generated by rotating the plane of motion (up to 40° in 12 azimuthal directions; Figure 3K; Methods). If grid-cell representations are preferentially aligned to the plane of motion, lattice fits should monotonically deteriorate as the candidate plane rotates away from the actual flight plane (Figure 3L). Consistent with this prediction, lattice fit quality was maximal on the true plane of motion and declined monotonically with increasing rotation angle (Figures 3M and S9). Together, these results suggest that grid-cell periodicity during structured flights is specifically aligned to the animal’s plane of motion, consistent with the mapping of a 2D toroidal manifold onto this behaviorally defined subspace.

This framework helps reconcile our findings with prior reports that did not detect periodic firing patterns of grid cells during movement in three-dimensional environments, where neural activity was analyzed without reference to the animal’s specific movement geometry^17,18^. It also argues against a simpler columnar organization, in which grid-cell firing would be referenced to the horizontal plane independently of the movement path, rather than to the animal’s plane of motion, as previously suggested for rodents moving on vertical pegboards and helical structures^31^. Although columnar and flight-plane-specific models can make similar predictions during structured flights restricted to a single plane, they diverge during unstructured flights spanning multiple planes of motion (Figure S10A). Consistent with a flight-plane-specific mapping, pooled unstructured flights showed significantly weaker plane-wave organization of toroidal phase maps (Figures S10B and S10C; mean MVL difference = 0.20 ± 0.03, mean ± s.d.; *P* < 0.001; Methods), and single-cell firing patterns were less consistent with a hexagonal lattice than during structured flights (Figures S10D and S10E; *P* = 2.32 × 10^−46^, *W* = 2618, *n* = 332; two-sided Wilcoxon signed-rank test). Similarly, apparent grid periodicity decreased monotonically as increasing number of distinct flight paths, occurring on different planes, were pooled together (Figures S10F and S10G; Methods). This reduction was consistent with simulations of flight-plane-specific grid cells in which mixing trajectories reduced observable periodicity despite each individual flight being periodic by construction (Figures S10H and S10I; Methods). Finally, toroidal latent-variable models trained on structured flights generalized readily to held-out unstructured behaviors (Figure S11; Methods), suggesting that reduced apparent spatial periodicity does not reflect a loss of the underlying toroidal population dynamics, but may instead arise from combining trajectories across different planes of motion.

Together, these results demonstrate that grid-cell representations in freely flying bats are well described by a two-dimensional toroidal manifold mapped onto the plane of motion of individual trajectories, providing a parsimonious solution for how a two-dimensional neural code can support navigation in a three-dimensional space.

## Discussion

Our findings show that grid cells retain periodic spatial firing patterns during three-dimensional movement and provide evidence for how the interplay between population dynamics and behavioral specialization gives rise to these activity patterns. At the single cell level, prior studies in rodents^18^ and bats^17^, using volumetric analyses of movement through 3D environments, reported irregular or locally ordered firing fields without global periodicity. Our results suggest that this apparent loss of periodicity can be reconciled by considering the ethological geometry of movement: bats do not sample 3D space uniformly but often navigate along locally planar trajectories. By allowing bats to express self-selected flight trajectories and analyzing neural activity in the coordinate frame of those trajectories, we reveal robust periodic grid-cell activity, highlighting the importance of considering not only the environments animals occupy, but also the species-specific behaviors they naturally express within them^32^. At the neuronal ensemble level, continuous attractor network (CAN) models^1,6–9^ have been successful in describing grid cells during movement in one- and two-dimensional environments, where population activity evolves on a low-dimensional toroidal manifold^10–16,23^. Here, we show that the activity of co-modular grid-cells in bats navigating in three-dimensional space exhibits topological signatures consistent with an underlying toroidal network organization, in line with experimental observations in rodents moving in one and two-dimensional environments^10–16^. Together, these results indicate that, despite their distinct mode of navigation, bats rely on similar entorhinal circuit principles as terrestrial mammals, supporting a unified, cross-species framework in which grid-cell activity reflects a shared computational mechanism across behavioral dimensions.

The central question therefore shifts from whether grid-cell periodicity, and the underlying toroidal population manifold, exist in three-dimensional space, to how a two-dimensional neural representation can support navigation through a three-dimensional world. Our results suggest that this is achieved by aligning grid-cell representations to behaviorally relevant subspaces. This framework helps reconcile a set of findings that have been difficult to integrate. In rodents, grid-cell firing can follow the geometry of locomotor surfaces that structure the animal’s motion: fields become vertically elongated on pegboards and helical tracks^31^, align to steeply sloped terrain^33^, and are altered during wall climbing^34^. Yet volumetric studies in rats and flying bats reported irregular^18^ or locally ordered^17^ firing fields without global periodicity, raising the possibility that grid structure breaks down when movement is no longer constrained to a surface. Our findings suggest a different resolution: during unconstrained 3D navigation, grid-cell periodicity and toroidal organization are preserved, but this structure becomes apparent only when interpreted relative to the animal’s plane of motion.

Consistent with this view, natural flight trajectories in bats, extending from the laboratory into the wild and across spatial scales, are well described by planar subspaces embedded within three-dimensional space, providing a natural behavioral substrate onto which grid activity can be mapped. Such a solution is also consistent with a broader principle of biological design: evolution often repurposes existing implementations rather than constructing entirely new ones^35^. In the case of grid-cells, such repurposing may reduce the need for higher-dimensional representations, which can carry substantial biological and computational costs^36,37^. Instead, a two-dimensional neural sheet may support behavior in three-dimensional environments by aligning to behaviorally relevant planes of motion. This suggests a tight correspondence between neural dynamics and ethological constraints, where the two-dimensional toroidal manifold and the planar structure of movement may reflect mutually compatible solutions that jointly enable efficient navigation in three-dimensional environments. More broadly, this provides a parsimonious account for how a common grid-cell architecture can be deployed across the mammalian tree despite substantial differences in navigational strategies (Figure S12)^27,28,38–40^. By aligning an evolutionarily conserved two-dimensional neural manifold to the ethologically relevant movement subspaces of each species, the brain may support three-dimensional navigation using a shared neural architecture rather than species-specific higher-dimensional solutions.

Finally, these findings highlight the importance of considering the ethological context of natural behavior when assessing neural computations. Neural circuits are shaped by evolution to support species-typical behaviors, and experimental conditions that restrict or distort those behaviors can obscure the computations they implement^32,41–45^. This is particularly important for naturalistic spatial behaviors, where the structure of the animal’s self-generated movement can determine whether an underlying neural code is apparent. Here, grid-cell periodicity in three-dimensional environments becomes evident only when neural activity is interpreted in the context of natural flight trajectories. Experimental paradigms that minimize external intervention, including human presence that can shape animal behavior and influence neural activity in the hippocampal formation^46^, may therefore better reveal the behavioral structure for which these circuits evolved. More generally, understanding brain function requires not only precise measurement of neural activity and behavior, but also experimental paradigms that preserve the natural structure of the behaviors these computations evolved to subserve.

## Material and Methods

### Subjects

Neural recordings were performed in a total of three adult male Egyptian fruit bats (*Rousettus aegyptiacus*; ~153-168g body weight). A total of 13 sessions (3-6 sessions *per* bat) were collected, during which neural activity was recorded wirelessly with a Neuropixels 1.0 (NP1.0) probe during aerial foraging. All animals were housed in a temperature- and humidity-controlled room. Implanted animals were single housed after implant surgery. Housing room lights were maintained on a reverse light cycle (12 h lights on, 19:00–07:00; 12 h lights off, 07:00–19:00). All experiments were performed during their awake hours (dark cycle) at the same time of the day. All experimental procedures were approved by the Institutional Animal Care and Use Committee of the University of California, Berkeley.

### Aerial foraging

All neural recordings during aerial foraging were performed in a large flight enclosure^19^ (5.6 m × 5.2 m × 2.5 m). During training and recording sessions, bats were mildly food-restricted at >85% of their baseline weight. Bats were trained for 7-14 days prior to performing neural recordings through daily (120 min) sessions in which the bats were allowed to freely obtain pureed fruit reward from automated feeders at designated locations in the enclosure. All trainings and recordings were performed without experimenters inside the flight enclosure to avoid confounds due to the presence of humans^46^. Recording sessions lasted between 80 and 150 minutes.

The flight enclosure is acoustically, electrically and radio-frequency-shielded, and equipped with high-precision lighting control. The room ceiling and walls were covered with acoustic foam to minimize acoustic reverberations and to dampen noise from outside the room. The walls and floors were covered with an additional layer of acoustically absorbing black felt to protect the acoustic foam from being damaged, and to provide a perch-able substrate for the bats. A set of 16 motion-capture cameras^19,20^ (Raptor-12HS, Motion Analysis) was used to track the 3D spatial position of the bats with millimeter-resolution. Three reflective markers, asymmetrically arranged on the neural recording headstage on the head of the bat, was tracked by the motion capture cameras at a frame rate of 120 Hz using commercially available software (Cortex-64; Motion Analysis). Two automated feeders, which dispense a pureed fruit reward, were positioned on the wall at one end of the room. Reward was triggered by an infrared beam break sensor that is interrupted when a bat landed on the feeding platform. Each feeder was independently controlled by an Arduino (Uno Rev3) and Adafruit Motorshield (1438; Adafruit) which interfaced with a computer outside of the experimental room. Reward probability (0.2 – 0.8) and amount (0.1-0.2 mL) were adjusted by the experimenter to fine tune the bat’s behavior. All recording sessions were conducted under uniform illumination (luminance level 5 lux).

All recording systems (position tracking and neural recordings) were synchronized by ground truth periodic clock pulses generated by a Master-9 device (A.M.P.I.) that served as the central clock for the experiments. On-board accelerometer data were acquired at 500 Hz by the neural recording headstage in parallel with the neural data.

### Surgery

Probe implants were performed in two stages, separated by 7-10 days: (i) implantation of the training cone, and (ii) insertion of Neuropixels 1.0 probes. The surgical procedure was performed as previously described in detail^21^.

#### Implantation of the cone

Anesthesia was performed using an injectable cocktail of ketamine, dexmedetomidine and midazolam. The bat was then placed on a stereotaxic apparatus (Model 942; Kopf) and provided with a continuous supply of oxygen. Anesthesia was maintained via injections of a cocktail of dexmedetomidine, midazolam, and fentanyl (~one dose per hour). Anesthesia depth was monitored continuously by the bat’s breathing rate and by a toe pinch reaction test. Body temperature was continuously monitored using a rectal temperature probe and maintained at approximately 35°C. Each Neuropixels probe was grounded using a stainless-steel wire (0.008 in. coated; A-M Systems) soldered to a ground screw (19010-00; FST), which was inserted into the frontal plane of the skull. Four shorter bone screws (M1.59 mm stainless steel) were inserted into the skull to further strengthen the attachment of the implant. A 1 mm circular craniotomy was made at the probe insertion point (at approximately 0.5 mm anterior to the transverse sinus and 4.9 – 5.7 mm lateral to the midline). The craniotomy was sealed with a biocompatible elastomer (Kwik-Sil; World Precision Instruments). At the end of the surgery, reversal agents were injected to counteract the dexmedetomidine and midazolam. An oral analgesic (Metacam; Boehringer Ingelheim) was administered after the bat has fully awoken from the anesthesia. Analgesics (3 days) and antibiotics (7 days) were provided daily post-op until full recovery. Behavioral training was resumed after full recovery from surgery (about four days). Implant weight was gradually increased during behavioral training to allow the bat to adapt to the full implant.

#### Insertion of Neuropixels 1.0 probes

Prior to probe insertion, each Neuropixels 1.0 probe (NP1) was sharpened at a 20-30° angle for 15 minutes using a Microgrinder (EG-45; Narishige), and a single stainless-steel wire (0.008 in. coated; A-M Systems) was soldered to the ground and reference of the probe. The probe insertion procedure follows the same general surgical practice as described above and previously^21^. After the bats were anesthetized and placed in a stereotaxic apparatus, the probe shank was coated with fluorescent dye (CM-DiI; Invitrogen C7001) and inserted into the target craniotomy at rate of ~10-20 um/sec at a pre-calculated angle. The initial insertion angle was calculated for the exact craniotomy location of each bat individually (between 20 – 40 degree azimuthal angle from the sagittal plane, and 15-25 degree pitch angle from the dorsal-ventral axis, such that the probe travels from posterior to anterior, medial to lateral). The probe was inserted to the maximum depth at which visible bending of the probe shank was observed (skull depth). If necessary, the probe was retracted and re-inserted following the above procedure with incremental changes in the insertion angle (10 degree azimuth and 2 degree pitch per step) until a desired skull depth of 6000-6500 um was measured. On the final insertion, the probe was retracted by 150-300 um to prevent contact with the skull during recordings. The probe was then cemented in place using dental acrylic and the ground wire of the probe was connected to the pre-existing ground screw. A new 3D printed protective cone was positioned and cemented to the skull and the bat was awoken using reversal agents to counteract the dexmedetomidine and midazolam.

### Electrophysiology data acquisition, pre-processing, and spike sorting

Recordings began one day after probe insertion (5 hours post-insertion for one bat) and were performed using a SpikeGadgets wireless NP1.0 headstage as previously described^21^. Channels were selected to record from dorsal MEC and was provisionally determined by a visible increase in spiking activity, typically around 4000 um depth (from probe tip) and subsequently verified histologically. Electrical signals (referenced to the ground screw) in the spike band (600 – 6000 Hz) and LFP band (0.5-200 Hz) were amplified 1000x – 1500x and 250x respectively. Drift correction and spike sorting was done automatically using Kilosort4^48^. All units labelled by Kilosort4 as *good* were then manually curated in Phy^49^. Rastermap^47^ visualization of population activity was computed with the following parameters: 10 ms time bins, ‘n_clusters’ = 96, n_PCs = 128, ‘time_lag_window’ = 50, and ‘locality’ = 0.3.

### Histology

At the end of the planned experiment sessions, bats were given a lethal overdose of sodium pentobarbital and perfused transcardially (200 ml phosphate buffered saline, 0.025M, pH = 7.4; 200 ml of fixative, 3.7% Formaldehyde in phosphate buffered saline). The probe implant was carefully removed, and the brain was dissected and stored in the fixative solution (3.7% Formaldehyde in phosphate buffered saline) for 1-2 days, then in a 30% sucrose solution in phosphate buffered saline overnight for cryoprotection. A microtome (HM450, Thermo Fisher Scientific) with a freezing stage was used to cut 50-μm sagittal section. Slices containing the medial entorhinal cortex were stained for DAPI and cover slipped with an aqueous mounting medium (ProLong Gold Antifade Mountant with DNA stain DAPI, Thermo Fisher Scientific). Fluorescent images of sagittal sections were acquired using an Axioscan Slide Scanner (Zeiss) and Neuropixels probe tracks were visualized and localized by CM-DiI fluorescence.

### Processing of position tracking data

Positional data recorded by the marker-based motion capture system (acquired at 120 Hz) was preprocessed^20^, to obtain continuous and smooth 3D positional data. Flights trials were segmented based on a velocity threshold of 0.5 ms^−1^, and 3D spatial trajectories during flight were clustered using hierarchical clustering^20,21^. In brief, flight trajectories were downsampled in time by a factor of 12, and clustered using agglomerative hierarchical clustering via Fréchet distance^21,50^. The linkage distance was selected to be the minimum threshold at which the silhouette score plateaued, where continued increase in linkage distance would result in less than a 0.05 increase in silhouette score. Flight clusters with at least 3 flights were defined as structured flight paths. All flights clusters with less than 3 flights were pooled together into the unstructured flight cluster.

### Defining stable multifield neurons

Stable multifield neurons were defined as those with (i) a minimum of 75 spikes on at least one structured flight path, (ii) an odd flight vs even flight spatial rate map Pearson correlation of at least 0.3, and (iii) a minimum of 4 firing fields on at least one structured flight path. The stability criteria of odd vs even spatial rate maps were constructed by splitting each structured flight cluster into odd and even trials. Odd trials from all structured flight clusters were combined into the odd group, and similarly for the even group. Spatial rate maps were then computed (see ‘Rate map estimation’) for the odd group and even group separately, using a bin size of 15 cm, gaussian kernel of 1.2 bins, and minimum occupancy threshold of 50 ms. To estimate the number of firing fields on a structured flight path, we first compute the spatial rate map on each structured flight path, then performed local maxima detection (using skimage function ‘peak_local_max’) of all peaks exceeding the mean rate map firing rate with minimum inter-peak distance of 30 cm. Subsequent analysis of each multifield neuron (see ‘Quantification of 1D periodicity’) was performed only on the subset of flight clusters in which it had sufficient spikes and multiple firing fields.

### Quantification of 1D periodicity

To quantify the periodic firing of grid cell responses on structured flight paths, spikes were binned into 10 ms time bins and neural activity was trial aligned to the time of take-off. Take-off time estimates were refined by finding the time-lag that minimized the mean squared error between each trial’s position trace and the cluster mean. Trial averaged firing rates were computed from the binned spike counts and smoothed with a 20 ms gaussian kernel. The autocorrelation of the trial-aligned firing rates was computed up to max lag equal to 50% of the trial length. Power spectral density (PSD) was computed from the trial-averaged firing rates using Welch’s method with window size equal to 80% of the trial length with 90% overlap. Cells were classified as significantly periodic if, on at least one flight path, both criteria are satisfied: (i) a PSD peak between 1Hz and 5 Hz that exceeds the 95^th^ percentile of shuffle (corresponds to the possible observed range of detectable grid spacings given the size of the flight enclosure, approximately 4 to up to 20 firing fields along a flight path), and (ii) peak in the autocorrelation with prominence greater than 0.1 between 0.2s and 1s time lag. The 95^th^ percentile PSD threshold was Bonferroni-corrected for the number of candidate flight paths each cell was evaluated on. Shuffled PSD distribution (*n* = 1,000 shuffles) was computed by applying a random circular shift to the spike train of each flight trial, thereby breaking the correspondence between spikes and position along flight paths while preserving the intrinsic spike train properties.

### Power spectral density clustering

Significantly periodic grid cells (as defined in ‘Quantification of 1D periodicity’), were pooled across sessions and clustered based on their power spectral density (PSD) along a representative flight cluster using agglomerative clustering with average linkage and cosine distances at threshold *ρ* = 0.5. For each cell, the representative flight cluster was chosen as the cluster with maximal fraction of total spectral power contained within a 0.3 Hz window around the largest PSD peak between 1 Hz and 5 Hz, and a minimum flight duration of 3 s. For visualization, a minimum cluster size threshold of 50 cells was used. The average grid spacing of each resulting cluster was estimated from the first positive-lag peak in the average cluster autocorrelation with prominence greater than 0.1. Autocorrelation heatmaps were then constructed by stacking each autocorrelation cluster sorted by their average grid spacing. For per-bat autocorrelation heatmaps, autocorrelation clusters were sorted by their average recording depth, and a minimum cluster size of 15 cells was used for visualization.

### Grid module ensemble detection

Detection of co-recorded grid module ensembles was performed using a spike-time-correlation-based method as proposed by Hermansen et al.^14^. In brief, we estimated the firing rate, $s_{i}(t)$, of each neuron by binning the spike times into 10ms time bins and smoothing with a 20ms gaussian kernel. We then computed the cross-correlation between each pair of co-recorded neurons across the full recording session (spanning all behavioral states, including flight and non-flight bouts):

$$c_{ij}(t)=\int_{0}^{T}s_{i}(\tau )s_{j}(t+\tau )d\tau ,$$

where T denotes the length of the full recording session. For flight versus non-flight comparisons (Figures 2A, 2B, and S3), the cross-correlations were performed using only in-flight time bins or non-flight time bins, respectively. Next, we computed the inverse, normalized cross-correlation matrix as:

$$c_{ij}=\frac{\underset{t}{min}{\left[c_{ij}(t),c_{ji}(t)\right]}_{0}^{\tau_{max}}}{\underset{t}{max}{\left[c_{ij}(t),c_{ji}(t)\right]}_{0}^{\tau_{max}}},$$

Where $\tau_{\text{max}}$ was chosen per recording between 100 – 200 ms (P1, P2, P3, R1: τ = 100 ms, Q1: τ = 200 ms). Finally, we computed the correlation distance between each row of **C^2^** to identify ensembles of neurons that have stable within-module relations, as grid cells should be consistently in phase or out of phase with each other. We then performed agglomerative clustering with average linkage using a distance threshold of *ρ* (P1: *ρ* = 0.375, P2: *ρ* = 0.475, P3: *ρ* = 0.475, Q1: *ρ* = 0.45, R1: *ρ* = 0.3). Ensemble detection code was based on implementations in the code repository by Hermansen et al^51^.

### Preprocessing of population activity

Preprocessing of population activity for persistent cohomology was performed as similarly described in previous studies in rodents^13,14^. Firing rates were computed by binning spikes into 10 ms time bins and smoothed with a 20 ms gaussian kernel to obtain population activity vectors (firing rate of each neuron in an ensemble). The population activity was temporally downsampled by a factor of 2 for in-flight time bins. Non-flight bouts were more heavily downsampled in time to reduce computational burden due to the large amount of time bats spend resting in a session (P1: 15, P2: 20, P3: 20, Q1: 20, R1: 10) .The firing rate of each neuron was z-scored relative to baseline, and the population activity vectors were projected into a 6D PCA subspace as done previously^13,14^. The population activity vectors were sorted by total activity, and the top fraction of time bins was selected to remove time bins with low or zero activity (Flight bouts, P1: 0.85, P2: 0.65, P3: 0.8, Q1: 0.6, R1: 0.85; Non-flight bouts, P1:0.65, P2: 0.75, P3:0.7, Q1: 0.45, R1:0.45).

Next, we performed a radial downsampling algorithm as proposed by Hermansen et al.^14^ to further reduce the size of the point cloud while preserving the spread of the data. In brief, the Euclidean distance of the point cloud to the point with maximal total activity (initial landmark point) was computed. Points with radial distance less than *ϵ* were then discarded and the point with the furthest distance to all previously chosen landmarks was picked as the next landmark point, and the process was repeated until no point remains within a radius of *ϵ* ϵ around landmark points (Flight bouts, P1: ϵ=0.25,P2:ϵ=0.05,P3:ϵ=0.05,Q1:ϵ=0.3,R1:ϵ=0.01; Non-flight bouts, P1:ϵ=0.4,P2:ϵ=0.15,P3:ϵ=0.15,Q1:ϵ=0.35,R1:ϵ=0.05 ). Radial down sampling preserves the point cloud shape but is prone to outliers, thus a second density weighted down sampling procedure was performed as described previously^13,14^ to reduce outliers and yield the final point cloud with desired size. In brief, the neighborhood strength of each point to its k nearest neighbors was defined as $m_{ij}=exp\left(-\frac{d_{ij}}{z_{i}}\right)$, where $d_{ij}$ is the cosine distance between points i and point j, and the normalization factor *z_i_* was chosen such that $m_{ij}$ sums up to ${log}_{2}(k)$, with k=1000. The symmetrical neighborhood strength is defined as $M_{ij}=m_{ij}+m_{ji}-m_{ij}\cdot m_{ji}$. This down sampling procedure was iteratively repeated until the final N points were chosen by picking at each step the point with maximal neighborhood strength (Flight bouts, P1: 1800, P2: 1200, P3: 1200, Q1: 1500, R1: 1300; Non-flight bouts, P1: 1650, P2: 1350, P3: 1650, Q1: 1500, R1: 1350). Finally, the negative logarithm of the downsampled point cloud neighborhood strengths was computed to yield a fuzzy distance matrix, which is then provided to the Ripser^52,53^ implementation for persistent cohomology computations.

### Persistent cohomology

To quantify the topological features in co-modular grid cell population activity (see ‘Preprocessing of population activity’), we used persistent cohomology, a topological data analysis technique previously used successfully for quantifying head direction ring topology^25,26,54^ and grid cell toroidal topology^13,14^. Persistent co-homology is closely related to persistent homology (described below), yields identical persistence barcodes, but is computationally efficient and enables cohomological decoding from computed cocycles^55^. The detailed theoretical framework of persistent cohomology is as described elsewhere^13,14,55^. In brief, a ball of radius r is placed at each point in the point cloud. As r is increased from zero, overlapping spheres merge, building up a sequence of connected shapes (a filtration) in which topological features such as connected components (0-d holes), circular features (1-D holes), and cavities (2-D holes) emerge at some radius, and is eventually filled. The range of radii at which a topological feature is present is denoted as bars in the persistence barcode, with longer bars indicating more robust topological features. Persistent cohomology computations were performed with Ripser^52,53^.

To confirm if the observed lifetimes of topological features exceed what is expected by chance, a random circular shift in time was applied to the activity of each neuron, and the topological lifetimes of each shuffle (*n* = 500) was computed using persistent cohomology following the same procedure as described above. To verify that the observed toroidal signature is above chance, we compared the observed barcode against toroidal signatures obtained in shuffled point clouds. Significance criterion of H0, H1, and H2 were set to the 95^th^ percentile of the longest finite H0 bar, the second longest H1 bar, and the longest H2 bar, respectively.

### Cohomological decoding of toroidal coordinates

Toroidal coordinates were decoded from the internal population dynamics via cohomological decoding introduced by de Silva et al.^55^, which has been applied previously for the study of head direction and grid cell population activity^13,14,25^. The implementation used here, described in brief below, is based on the published methods and code from Gardner et al.^13^ and Hermansen et al.^14,51^. Persistent cohomology was computed on the downsampled point cloud of grid cell activity (see ‘Preprocessing of population activity’ and ‘Persistent cohomology’). Circular-valued coordinates were then decoded for each of the two longest-lived H1 bars from their cocycle representatives by lifting to integer coefficients and smoothing at a filtration scale τ = b + 0.99·(d — b), where b and d denote the birth and death time of the second longest H1 bar. To improve robustness to non-uniform sampling of the underlying manifold, smoothing was performed under successively higher L^p^ norms (p = 2, 4, 6, 10, 20) and finally under the L^∞^ norm, following procedures established in Paik & Park^56^. Interpolation of toroidal coordinates to time points beyond that of the downsampled point cloud, both within and across behavioral states, was done by computing for each cell, the distribution of toroidal coordinates weighted by the cell’s z-scored firing rate. Toroidal coordinates of any given population activity vector were then computed as the center of mass of the per-cell distributions weighted by each cell’s activity in the query activity vector.

### Rate map estimation

#### Spatial rate map

To compute 2D spatial rate maps, spike and bat positions were binned into 10 cm x 10 cm bins. The binned spike counts and binned occupancy were then smoothed with a gaussian kernel (σ= 1.5 bins). Spatial rate maps were then estimated by dividing the smoothed binned spike counts by the smoothed binned occupancy, and all bins with occupancy <30 ms were excluded.

#### Toroidal rate map

Toroidal rate maps were estimated from the spike counts and decoded toroidal coordinates (see ‘Cohomological decoding of toroidal coordinates’) per 10 ms time bin. The full toroidal phase space was divided into 18° x 18° bins, and toroidal phase occupancy was computed by binning the decoded toroidal coordinates. Toroidal phase spike counts were tabulated by cumulating the number of spikes that occurred in each toroidal phase bin. Before smoothing, each row in the toroidal phase occupancy and spike count maps was rolled horizontally by half its vertical index to account for the 60° angle between the toroidal axes. The sheared maps were then tiled into a 3x3 grid, smoothed with a gaussian kernel (σ = 1.5 bins), and cropped to the central tile (following similar procedures used in rodents^13,14^). The toroidal rate map was then computed as done for spatial rate maps by dividing the smoothed spike counts by the smoothed occupancy maps. For visualization, the axes of the toroidal rate map were sheared by 15°.

Pairwise similarity between spatial rate maps or toroidal rate maps is measured with the Pearson correlation between flattened rate maps on shared occupancy bins. Correlation between pairwise spatial similarity and pairwise toroidal similarity was quantified by Pearson correlation in comparison to a shuffle distribution generated by randomly permuting the cell identities (*n* = 1,000 shuffles).

### Toroidal phase map estimation

Toroidal phase maps were computed by binning the toroidal coordinates at each bat position (projected onto the flight plane, unless otherwise noted) into 10 cm x 10 cm bins and taking the circular mean of the toroidal coordinate within each bin. The resulting phase maps were smoothed by applying a gaussian kernel (σ = 0.6 bins) to the sine and cosine projections of the phase and recombining them with arctan2. A minimum occupancy threshold of 10 ms was used. Cosine-projected toroidal phase maps, used for visualizations only, were computed by taking the cosine of the smoothed angular map and applying an additional gaussian kernel (σ = 0.6 bins) smoothing step.

### Stability of toroidal tuning

To assess the stability of grid cell tuning to the underlying toroidal manifold across behavioral states, a separate toroidal parameterization was computed using flight bouts and non-flight bouts (see ‘Persistent cohomology’ and ‘Cohomological decoding of toroidal coordinates’). Toroidal rate maps during flight bouts were computed from the flight bout toroidal parameterization, and rate maps during non-flight bouts were computed from both (i) the same flight bout toroidal parameterization, or (ii) from a separate parameterization computed from non-flight time bins. When using the same flight-bout toroidal parameterization, the similarity between toroidal tuning during flight and non-flight is measured by the Pearson correlation between the toroidal rate maps. With separate toroidal parameterizations for flight and non-flight, correspondence between $\left(\phi_{1},\phi_{2}\right)$ obtained during flight and during non-flight is not guaranteed as the decoded coordinates may correspond to any two of the three possible axes of a hexagonal lattice and may also differ in choice of origin and orientation. Following previously described procedures^14^, we smoothed the decoded toroidal coordinates obtained from each parameterization in time with a gaussian kernel of 20 ms, then tested all possible $\left(\phi_{1},\phi_{2}\right)$ correspondences, sign flips, and phase shifts, and picked the one that yielded the minimum angular error between flight and non-flight parameterizations.

### Analysis of flight geometry

#### Flights in wild

We analyzed the geometry of Egyptian fruit bat flight during large-scale navigation in the wild using a publicly available GPS-tracking dataset from Egert-berg et al.^29,30^, which tracked free-ranging bats commuting in the wild between roosts and distant foraging sites. Position traces were preprocessed using a 15-sample median filter followed by a Gaussian filter (σ = 2 samples). Missing gaps of ≤3 samples were linearly interpolated. Initial flight segmentation was based on a velocity threshold of 10 km h^−1^, after which flight boundaries were refined by extending each segment (up to 25% of the initial segment length) to the nearest sample with altitude <5 m. Flights were then filtered for commute trajectories with total distance traveled ≥10 km and straightness index^27,30^ ≥ 0.7 where straightness index was defined as the ratio of the shortest path between flight endpoints to the actual path length. The cruise phase of each flight was defined as the portion of the trajectory between the first and last samples exceeding 75% of the maximum flight altitude. Ascend and descend phases were defined as the trajectory segments preceding and following the cruise phase, respectively. Flights with ascent or descent phases spanning <50 m altitude change, or cruise phases shorter than 5 km, were excluded from further analysis. Each flight phase was fit independently using piecewise planar models with K = 1 – 5 planes, optimized using a L1-loss objective. Constraints were imposed on allowable plane orientations (|roll| ≤45°, |pitch| ≤75°). For each K, piecewise variance explained was defined as:

$$VE(K)=1-\frac{\sum_{K}SSE_{⊥}}{\sum_{K}SST},$$

Where *SSE*_⊥_denotes the sum of squared residuals of each segment from its plane, and SST is the total sum of squares of each segment from its centroid. For K=1, this reduces to the standard variance explained by a single best-fit plane. A flight phase is defined to be well-fit by K planes if the piecewise variance explained is greater than 0.98.

#### Flights in laboratory

Following preprocessing of foraging flights collected in the laboratory (see ‘Processing of position tracking data’), the same piecewise plane fitting procedure described above was applied to each individual flight trial, including both structured and unstructured flight clusters. Flights were classified according to the minimum number of planes required to achieve piecewise variance explained > 0.98.

### Fitting of plane waves

To fit the toroidal phase maps on the flight plane with plane waves, we performed a grid search over all plane wave angles (0 to 180° in steps of 1°) and grid spacings (125 geometrically spaced steps between 0.6 and 2.5m). Optimal plane wave phase was computed analytically for a given angle and spacing. As $\phi_{1}$ and $\phi_{2}$ are offset by 60° or 120° for hexagonal grids, we fit the $\phi_{1}$ and $\phi_{2}$ phase patterns simultaneously, with a fixed angular offset between the plane waves for each. The mean vector length (MVL) of the angular error distribution was used as the fitting metric (higher MVL indicates better fit and is bounded between 0 and 1). The shuffle distribution was constructed by rolling each toroidal coordinate dimension by a random amount in time per flight trial, and repeating the same plane wave fitting procedure as described. For each module, a shared grid spacing was estimated by z-scoring the maximum MVL obtained at each spacing against that of the shuffled distribution and picking the spacing that gives the highest average MVL across structured flight clusters. Since $\phi_{1}$ and $\phi_{2}$ could correspond to plane waves either 60° or 120° apart, the fitting procedure was repeated for both angular offsets and the one that yielded the highest average MVL score on structured flights for each module was chosen. Significance of the plane wave fit for each module was assessed by the average MVL at the shared spacing relative to the 95^th^ percentile of the shuffle distribution (*n* = 1,000 shuffles). For structured versus unstructured plane wave fit comparisons, each structured flight cluster was fit without shared constraints across clusters for fair comparison with the independently fit unstructured flight cluster. The observed difference in structured versus unstructured MVL score was averaged across modules and evaluated relative to the 95^th^ percentile of the distribution of average MVL score differences from each shuffle (*n* = 1,000 shuffles).

### Fitting of hexagonal grids on planes of motion

To fit grid patterns on the plane of motion to grid cell activity, the observed spikes and bat positions were projected onto the flight plane. We performed an exhaustive grid search over the full landscape of grid spacing (75 geometrically spaced steps from 0.6m to 2.5m), orientation (0° to 60° in 1° steps), and xy phase (14 x 14 = 196 total phase bins tiling the full phase space). For each grid parameter combination, a candidate rate map was constructed with hexagonally arranged firing fields with the given grid spacing, orientation, and phase. Grid fields were modeled as Gaussian profiles with σ = 0.125 · *s*, where *s* is the grid spacing. Each flight cluster was fit independently using the Poisson log-likelihood of the observed spike positions as given by:

$$LL=\underset{i}{\sum }\text{log}\left(\lambda \left(x_{i}\right)\right)-\underset{t}{\sum }\lambda \left(x_{t}\right)\cdot \text{Δ}t,$$

where $\lambda \left(x_{i}\right)$ is the firing rate of the candidate grid pattern at the position of spike *i*, $\lambda \left(x_{t}\right)$ is the firing rate at the bat position at time t, and Δ*t* is the time bin size (10 ms). The shuffle distribution is constructed by applying random circular shift to each trial of a flight cluster and performing the same grid fit procedure as described above. The spacing of each cell was estimated by z-scoring the maximum log-likelihood at each grid spacing relative to that of the shuffle distribution at that spacing, and picking the shared grid spacing with the highest average z-scored log-likelihood across structured flight clusters. The total log-likelihood of observed spikes, evaluated at the shared grid spacing across structured flights, was z-scored against that of the shuffle distribution to obtain a z-scored log-likelihood grid fit metric. A grid fit was considered significant if its total log-likelihood exceeded the 95th percentile of the shuffle distribution (*n* = 200 shuffles).

Grid fitting on planes rotated from the plane of best fit was performed by projecting the spike positions onto each rotated plane of motion and applying the same fitting procedure as above. Near-straight flights were excluded, as they are largely invariant under rotations about their principal axis. For each cell, fitting on rotated planes was restricted to flight clusters on which it had a significant grid fit on the cluster’s plane of best fit (Figure 3M). To verify the effect was not due to selection of significant grid cells based on the best fit plane, the analysis was repeated using all flight clusters where the cell was significant on any of the rotated planes, Bonferroni-corrected for multiple comparisons across the 49 planes (Figure S9C).

Candidate grid cells were identified from the inter-spike-distance distribution of each unit. For each unit, the pairwise Euclidean distance between every spike from all trials of a flight cluster were binned into a 1D histogram (0–5 m, 5 cm bin size). The distribution was smoothed with a Gaussian kernel (σ = 1.5 bins) and peak normalized. Peaks were detected with the SciPy function ‘find_peaks’, using a minimum prominence of 0.2 and a minimum separation of 0.25 m. A unit was included if it satisfied: (i) ≥ 150 spikes emitted during flights; (ii) at least two periodic peaks greater than 0.5 m, with at least one below 2 m.

For comparisons between structured and unstructured flight clusters, we computed, for each cell, the difference in z-scored log-likelihoods (structured minus unstructured). Significance of the paired difference distribution from zero was assessed with a two-sided Wilcoxon signed-rank test.

### Grid cell periodicity on mixtures of flight planes

To quantify the effect of pooling together flights on different planes on the observable grid cell periodicity, we pooled together the structured flight clusters in all combinations and fit hexagonal grids to the resulting activity patterns (see ‘Fitting of hexagonal grids on planes of motion’). Grid fitting was performed on the top-down projected activity patterns (columnar model) in the room-frame. To determine if a plane-dependent grid code is sufficient to explain the observed reduction in periodicity when flights are pooled, we simulated grid cells from hexagonal grid patterns defined on the plane of motion of each flight trajectory (relative to the reference frame spanned by its first two principal components, or PCA-frame). Grid spacing, orientation, and phase of simulated grid cells were matched to the distribution of grid parameters obtained from grid fitting on real cells. The same combinatorial flight path pooling and grid fitting procedure was performed as above. As the unstructured flight cluster already contains diverse flight trajectories, it was included only in the separate ‘All’ group containing all flights in a session.

### Toroidal latent variable model

To rigorously assess whether the toroidal latent dynamics during structured flights with clear grid cell periodicity generalizes to that of unstructured flights despite the lack of periodicity, we adapted the toroidal latent variable model (LVM; Figure S11A) from the architecture and code provided by Bjerke et al^57^. In brief, the LVM consists of an encoder that maps population activity to a toroidal latent state, and a decoder that predicts neural activity from the latent state. The LVM is given by p(x,z)=p(x∣z)p(z), where *x* is the population activity vector and *z* = (*z*_1_, *z*_2_) is the toroidal latent state. The decoder *p*(*x*|*z*) models the activity of cell i as *x_i_* ~ *Poisson*(*g*(*z*, *u_i_*)), where (*g*(*z*, *u_i_*) is a Gaussian-like tuning curve centered at the cell’s preferred phase *u_i_*, with geodesic distances on the toroidal manifold. The latent prior factorizes as *p*(*z*) = *p*(*z*_1_)*p*(*z*_2_) with (*z*_1_, *z*_2_) each drawn from a wrapped Normal distribution. The encoder is a 1D convolutional neural network parameterizing the variational posterior *q*(*z*|*x*), allowing the model to be trained end to end by maximizing the evidence lower bound (ELBO). Using the LVM, we trained two models using 5-fold cross-validation: one on structured flights only and one on unstructured flights only. For each fold, each LVM was trained on 80% of its respective flight data and evaluated on the same held out 20% of unstructured flights (Figure S11B). Model performance was quantified by pseudo-R^2^, with a mean-firing-rate baseline as the null.

### Data Analysis

All analyses were conducted using custom code in Python and MATLAB (2024b, MathWorks). The following open-source packages were used: numpy (2.2.6), scipy (1.16.1), scikit-learn (1.7.2), ripser (0.6.12), rastermap (1.0), pytorch (2.9.1), kilosort4 (4.1.1). Computationally heavy workloads (‘Fitting of hexagonal grids on planes of motion’ and ‘Persistent Cohomology’) were performed on the Savio computational cluster provided by the Berkeley Research Computing program at the University of California, Berkeley.

### Statistical analysis

Sample sizes were not predetermined using formal methods; Sample sizes used were comparable to those of similar studies. Experiment sessions were not randomized, and experimental conditions were not blinded. All statistical tests were one-sided unless otherwise stated.

## Supplementary Material

1
