# Supplementary material for: A Two-Dimensional Grid-Cell Code for Three-Dimensional Navigation in Freely Flying Bats
Source: bioRxiv. 2026 Jun 11:2026.06.10.728358. Preprint. [Version 1] doi: 10.64898/2026.06.10.728358 (PMC13277914; doi:10.64898/2026.06.10.728358)
Supplement: Supplementary file 1 [file NIHPP2026.06.10.728358v1-supplement-1.pdf]

bioRxiv preprint doi: https://doi.org/10.64898/2026.06.10.728358; this version posted June 11, 2026. The copyright holder for this preprint (which was not certified by peer review) is the author/funder, who has granted bioRxiv a license to display the preprint in perpetuity. It is made available under a CC-BY 4.0 International license.

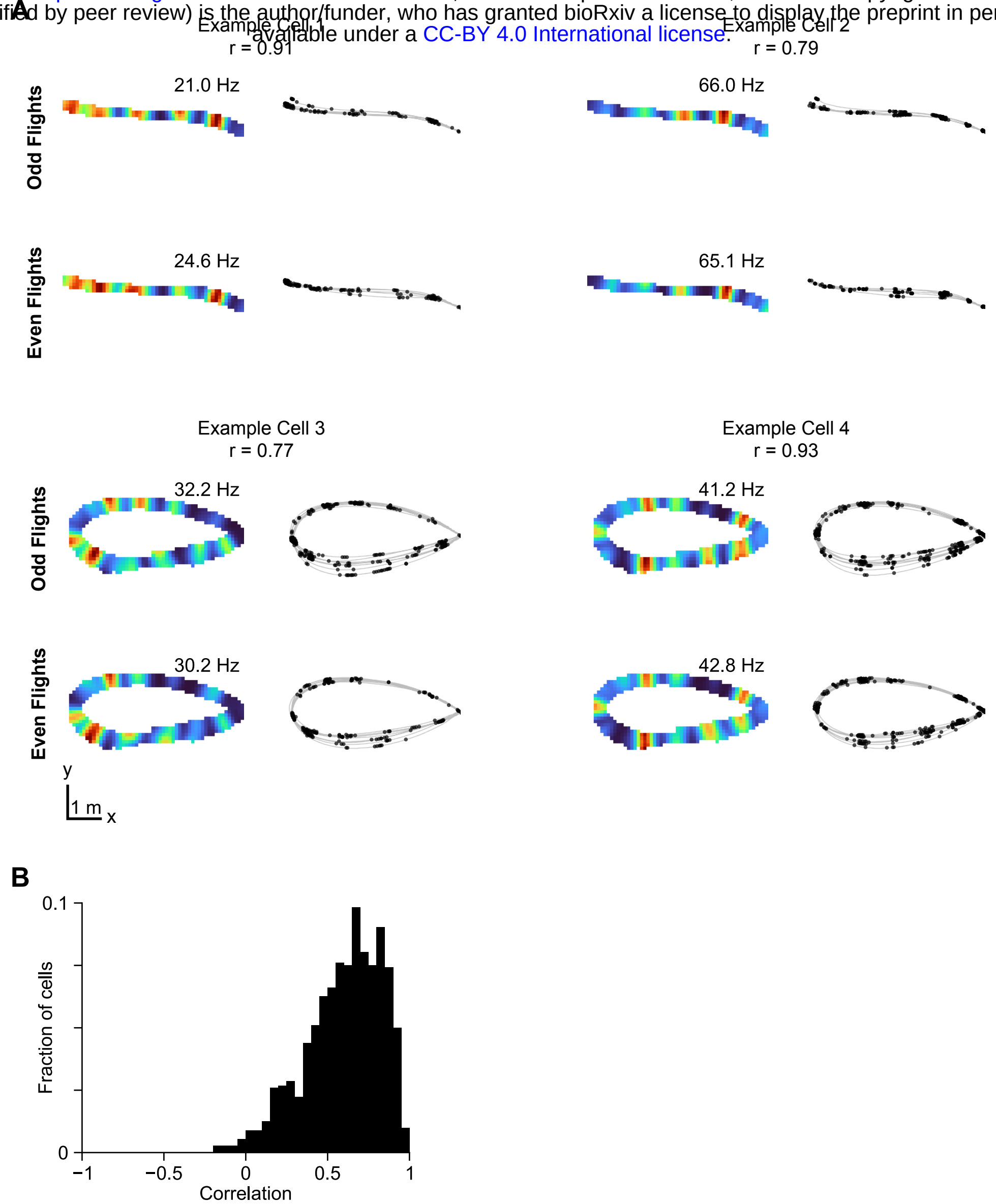

Figure S1. MEC neurons exhibit stable spatial firing fields across flights
(A) Rate maps and raw spike locations for four example MEC neurons along repeated flight trajectories, split by odd and even flights. For each example, occupancy-normalized firing-rate maps are shown on the left and spike locations (black) overlaid on flight trajectories (grey) are shown on the right. Peak firing rate for each rate map is indicated.
(B) Distribution of correlation between odd- and even-flights rate maps (Methods) across neurons.

bioRxiv preprint doi: https://doi.org/10.64898/2026.06.10.728358; this version posted June 11, 2026. The copyright holder for this preprint (which was not certified by peer review) is the author/funder, who has granted bioRxiv a license to display the preprint in perpetuity. It is made available under a CC-BY 4.0 International license.

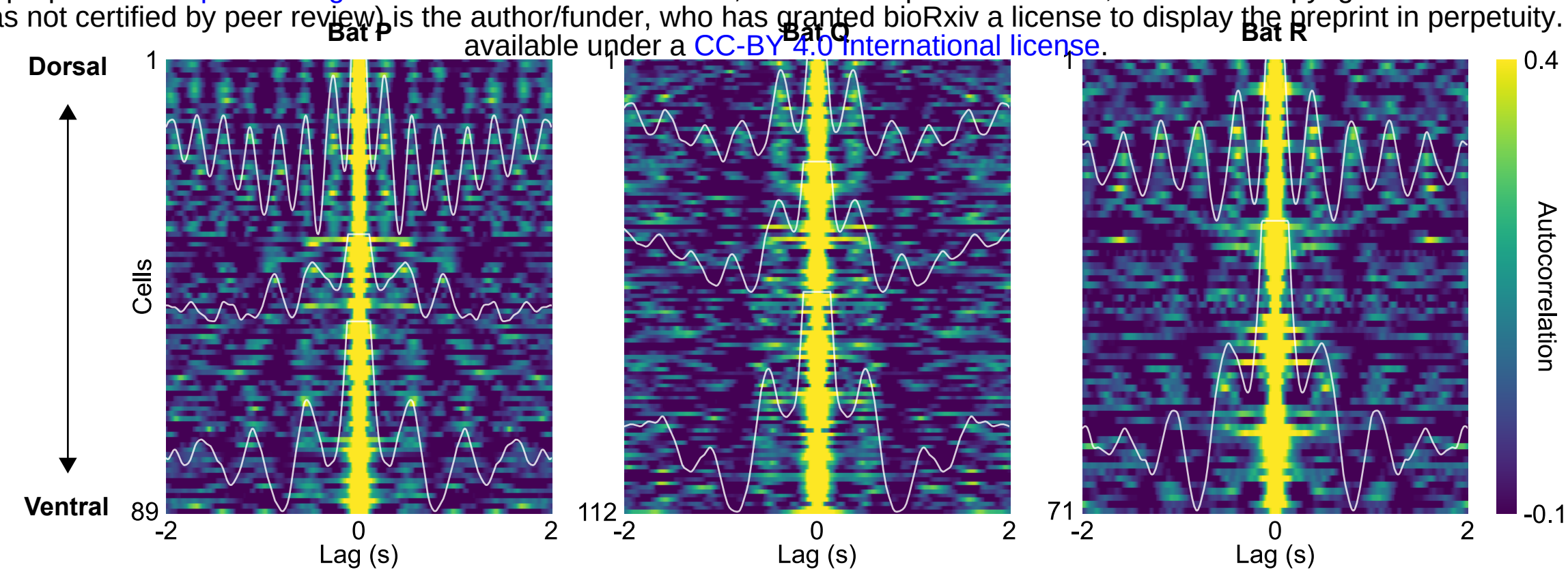

Figure S2. Topographic module-like organization of grid cells along dorso-ventral MEC axis

Time-lag autocorrelations of significantly periodic grid cells, shown separately for each bat and sorted from dorsal to ventral by mean cluster recording depth (Methods). Cells were clustered using the normalized power spectral densities (PSD) of their autocorrelations across all bats. White traces indicate the mean autocorrelation for each cluster. Only within-bat clusters containing 15 or more cells are shown.

bioRxiv preprint doi: https://doi.org/10.64898/2026.06.10.728358; this version posted June 11, 2026. The copyright holder for this preprint (which was not certified by peer review) is the author/funder, who has granted bioRxiv a license to display the preprint in perpetuity. It is made available under a CC-BY 4.0 International license.

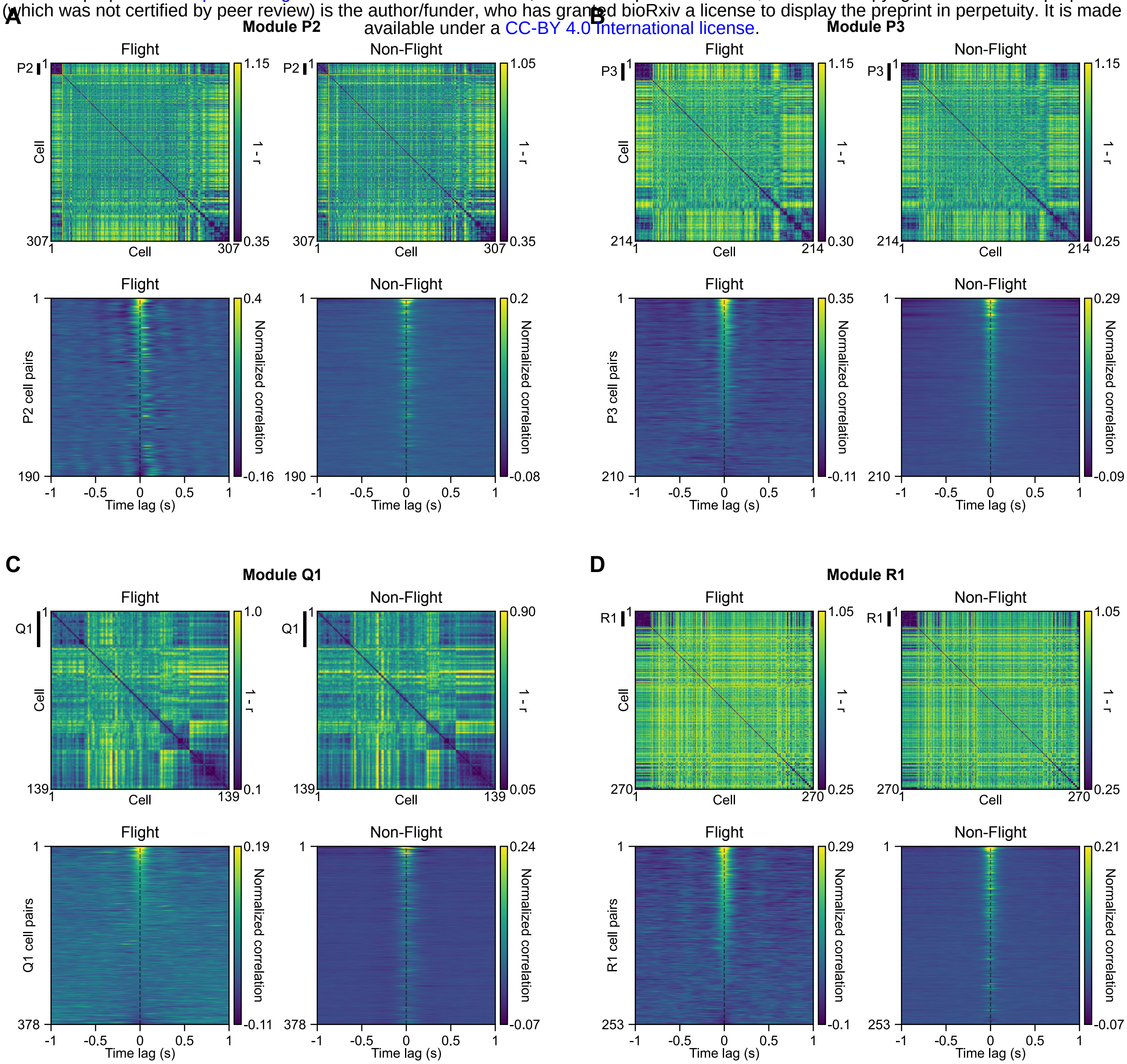

Figure S3. Stable pairwise spike-time correlations of co-modular grid cells across behavioral states

(A-D) Spike-time-correlation-based clustering (Methods) of four additional putative grid modules not shown in Figure 2A and 2B, computed separately during flight and non-flight bouts. For each module, top panels show correlation-distance matrices during flight (left) and non-flight (right) periods, clustered and sorted according to flight bouts. The same clustering and sorting were then applied to the corresponding non-flight matrix. Bottom panels show within-module pairwise normalized cross correlation during flight (left) and non-flight (right) periods, sorted by zero-lag correlation during flight bouts. The same sorting was applied to non-flight bouts. The cross-correlation of each cell pair was normalized by mean subtraction.

bioRxiv preprint doi: https://doi.org/10.64898/2026.06.10.728358; this version posted June 11, 2026. The copyright holder for this preprint (which was not certified by peer review) is the author/funder, who has granted bioRxiv a license to display the preprint in perpetuity. It is made available under a CC-BY 4.0 International license.

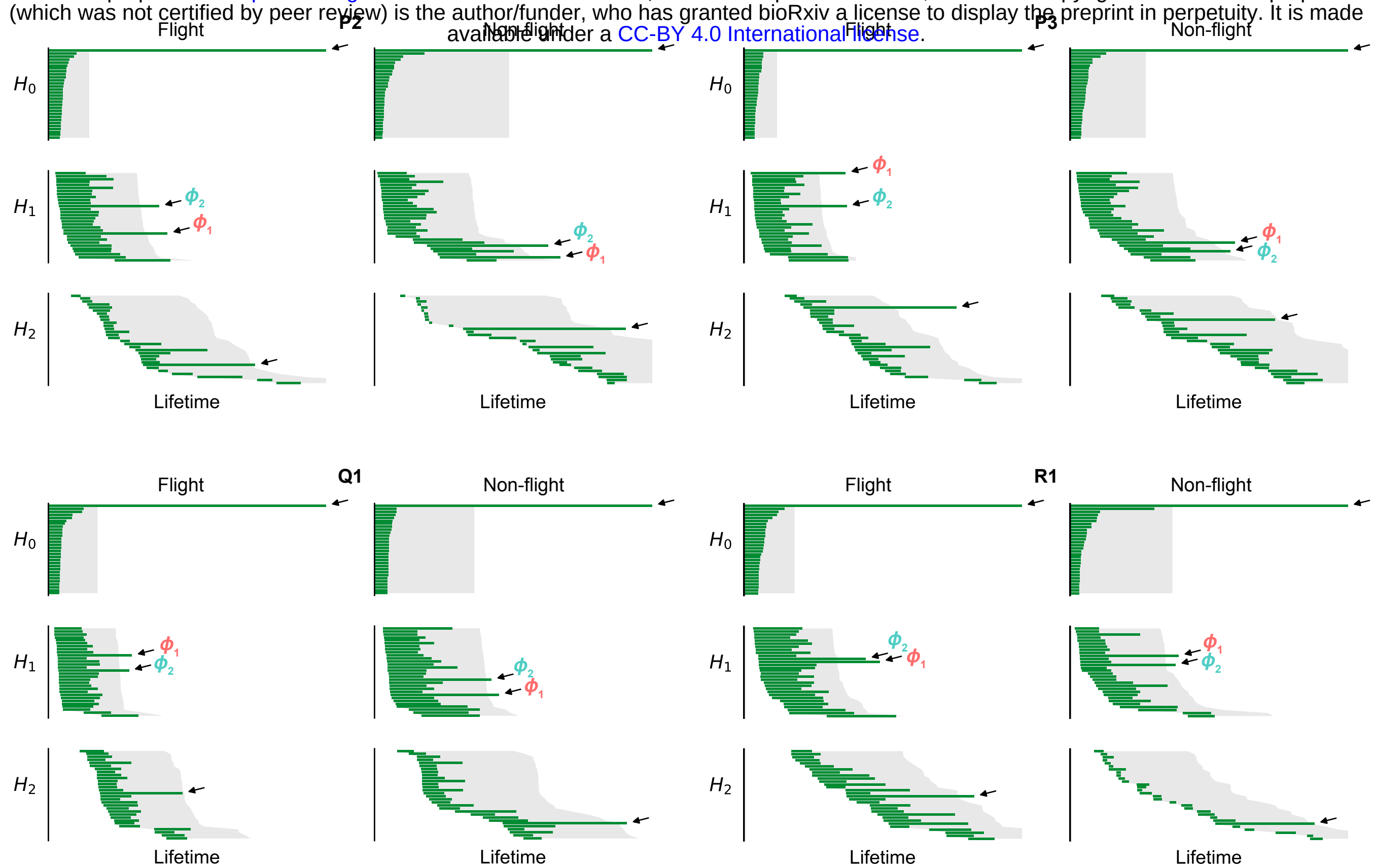

Figure S4. Persistence barcodes indicating toroidal topology of grid cell population activity
Persistence barcodes computed during flight (left) and non-flight bouts (right) for the four additional putative grid modules not shown in Figure 2C: P2, P3, Q1 and R1. Green bars indicate the lifetime of topological features in each homology dimension: connected components (H_0), circular features (H_1) and cavities (H_2). Arrows mark the prominent features, which are consistent with a toroidal topology. Grey shading indicates the 95th percentile of the shuffle distribution, constructed by circularly shifting each cell's spike train relative to the others. The test statistic was the longest bar for H_0 and H_2, and the second-longest bar for H_1 (Methods).

bioRxiv preprint doi: https://doi.org/10.64898/2026.06.10.728358; this version posted June 11, 2026. The copyright holder for this preprint (which was not certified by peer review) is the author/funder, who has granted bioRxiv a license to display the preprint in perpetuity. It is made available under a CC-BY 4.0 International license.

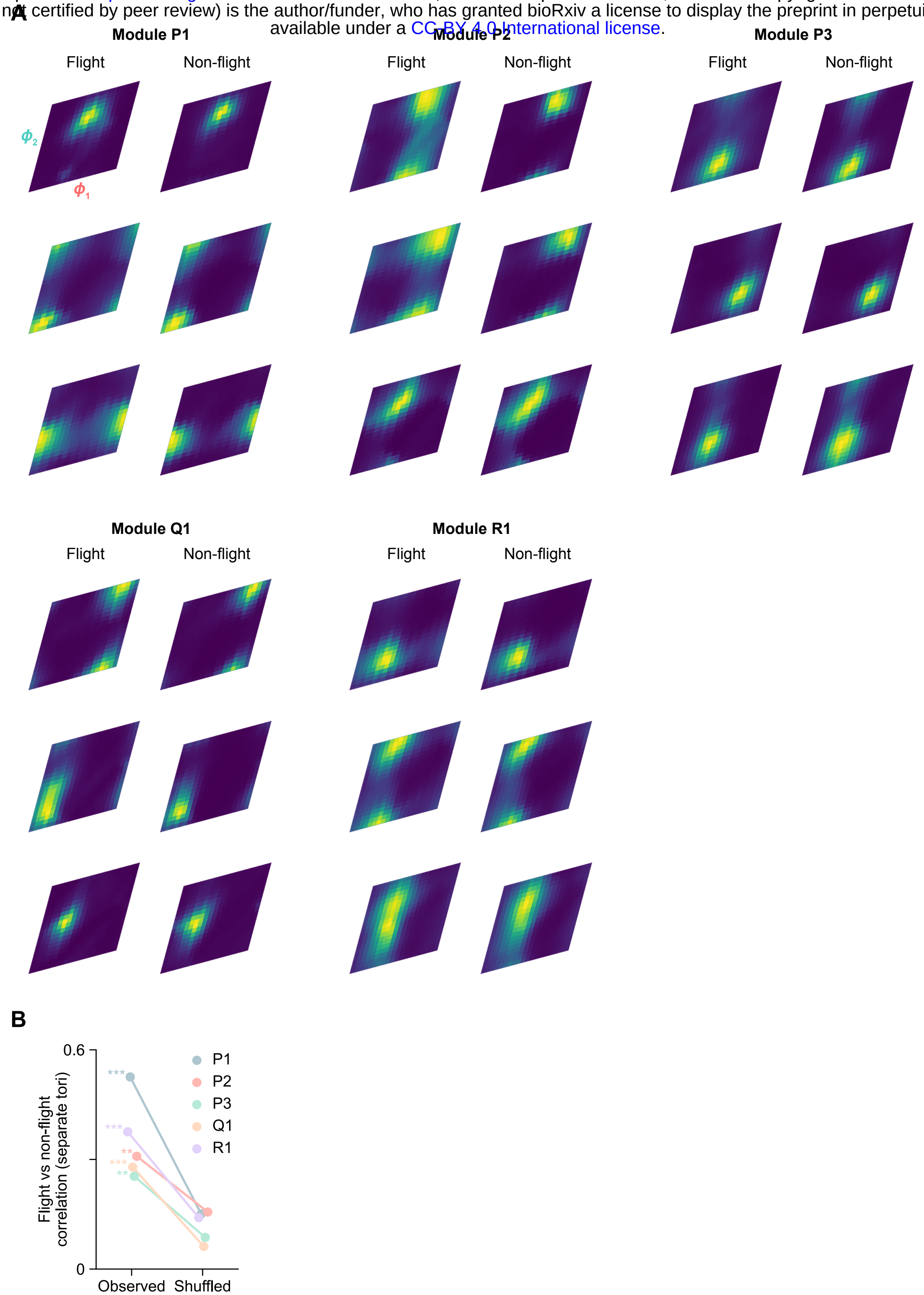

Figure S5. Toroidal rate maps are stable across flight and non-flight bouts
(A) Example toroidal rate maps of grid cells from each module during flight (left), and non-flight bouts (right). Rate maps are plotted in toroidal coordinates and normalized to the peak firing rate of each cell (Methods). **(B)** Pearson correlation between flight and aligned non-flight toroidal rate maps, computed using separate toroidal parameterizations for each behavioral state (Methods). Lines connect paired comparisons for each module, indicated by colors. $n = 20$ (P1), 20 (P2), 21 (P3), 28 (Q1), 23 (R1) cells. ***$P<0.001$; **$P<0.01$.

bioRxiv preprint doi: https://doi.org/10.64898/2026.06.10.728358; this version posted June 11, 2026. The copyright holder for this preprint (which was not certified by peer review) is the author/funder, who has granted bioRxiv a license to display the preprint in perpetuity. It is made available under a CC-BY 4.0 International license.

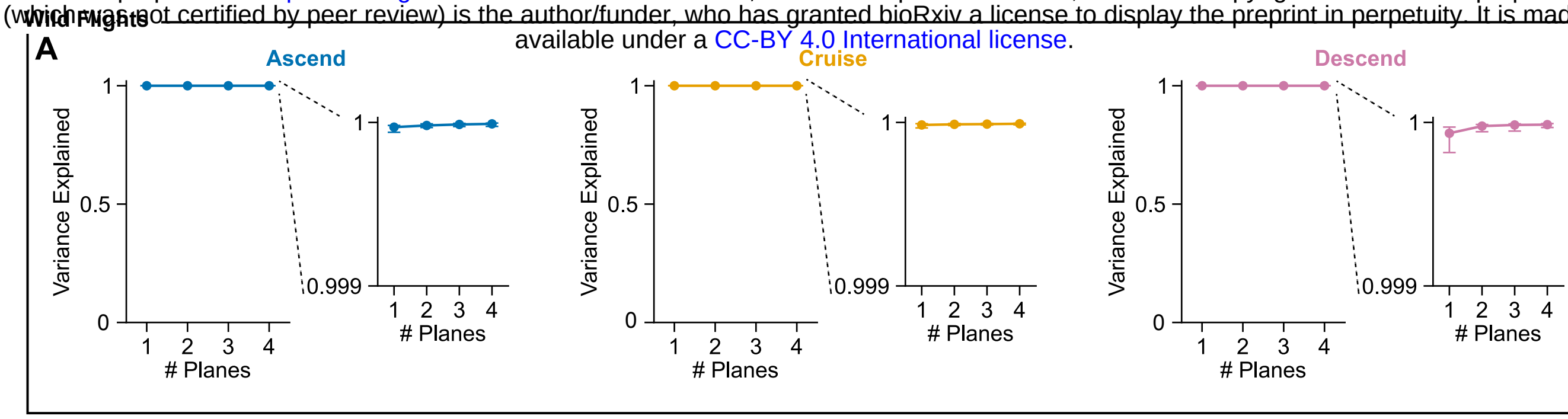

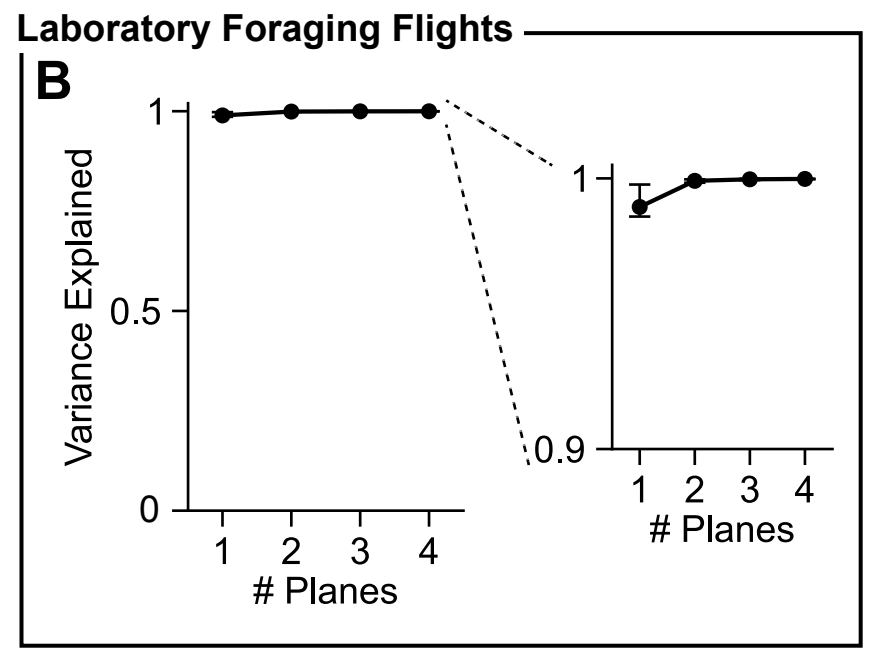

Figure S6. Quantification of planar flight structure in the wild and in the laboratory
(A) Median fraction of 3D positional variance explained by one or more fitted planes during ascend (left), cruise (middle), and descend (right) phases of wild commute flights. Error bars denote the interquartile range. Insets show zoomed views. **(B)** Median fraction of 3D positional variance explained by fitted planes as in a, for laboratory foraging flights. Error bars indicates the interquartile range. Inset shows zoomed view.

bioRxiv preprint doi: https://doi.org/10.64898/2026.06.10.728358; this version posted June 11, 2026. The copyright holder for this preprint (which was not certified by peer review) is the author/funder, who has granted bioRxiv a license to display the preprint in perpetuity. It is made available under a CC-BY 4.0 International license.

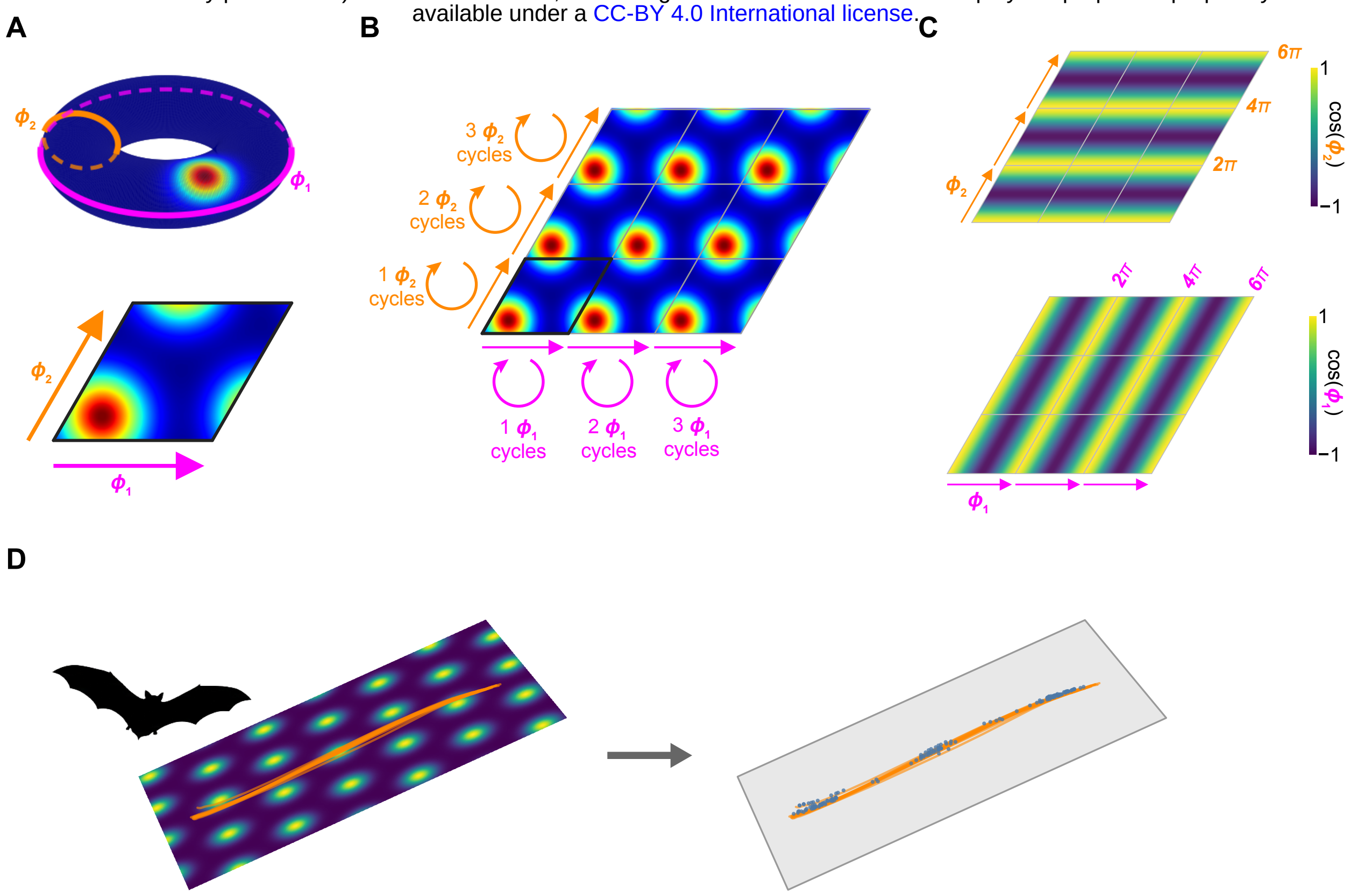

Figure S7. Conceptual illustration of mapping toroidal grid-cell representations onto two-dimensional space

(A) Top, schematic of a grid cell toroidal manifold defined by two circular coordinates, ϕ_1 (pink) and ϕ_2 (orange). Example tuning field denotes the activity of one grid cell at a specific ϕ_1 and ϕ_2 position on the torus. Bottom, the primitive rhombic unit cell of a two-dimensional hexagonal lattice. The torus is equivalent to this unit cell under periodic boundary conditions, with ϕ_1 and ϕ_2 spanning the two edges of the rhombus. **(B)** Periodic tiling of two-dimensional space by the rhombus unit cells generates a hexagonal grid pattern. ϕ_1 and ϕ_2 repeat along the principal axes of the hexagonal lattice, along the edges of the rhombii. **(C)** Toroidal phase coordinates (ϕ_1, ϕ_2) form plane-wave patterns across space, corresponding to the tiling of the rhombus unit cell. **(D)** Schematic of a bat's flight trajectory (orange) intersecting hexagonal grid fields on its plane of motion (left). Under this mapping, firing (blue dots) along the flight trajectory is predicted to be consistent with the underlying hexagonal lattice (right).

bioRxiv preprint doi: https://doi.org/10.64898/2026.06.10.728358; this version posted June 11, 2026. The copyright holder for this preprint (which was not certified by peer review) is the author/funder, who has granted bioRxiv a license to display the preprint in perpetuity. It is made available under a CC-BY 4.0 International license.

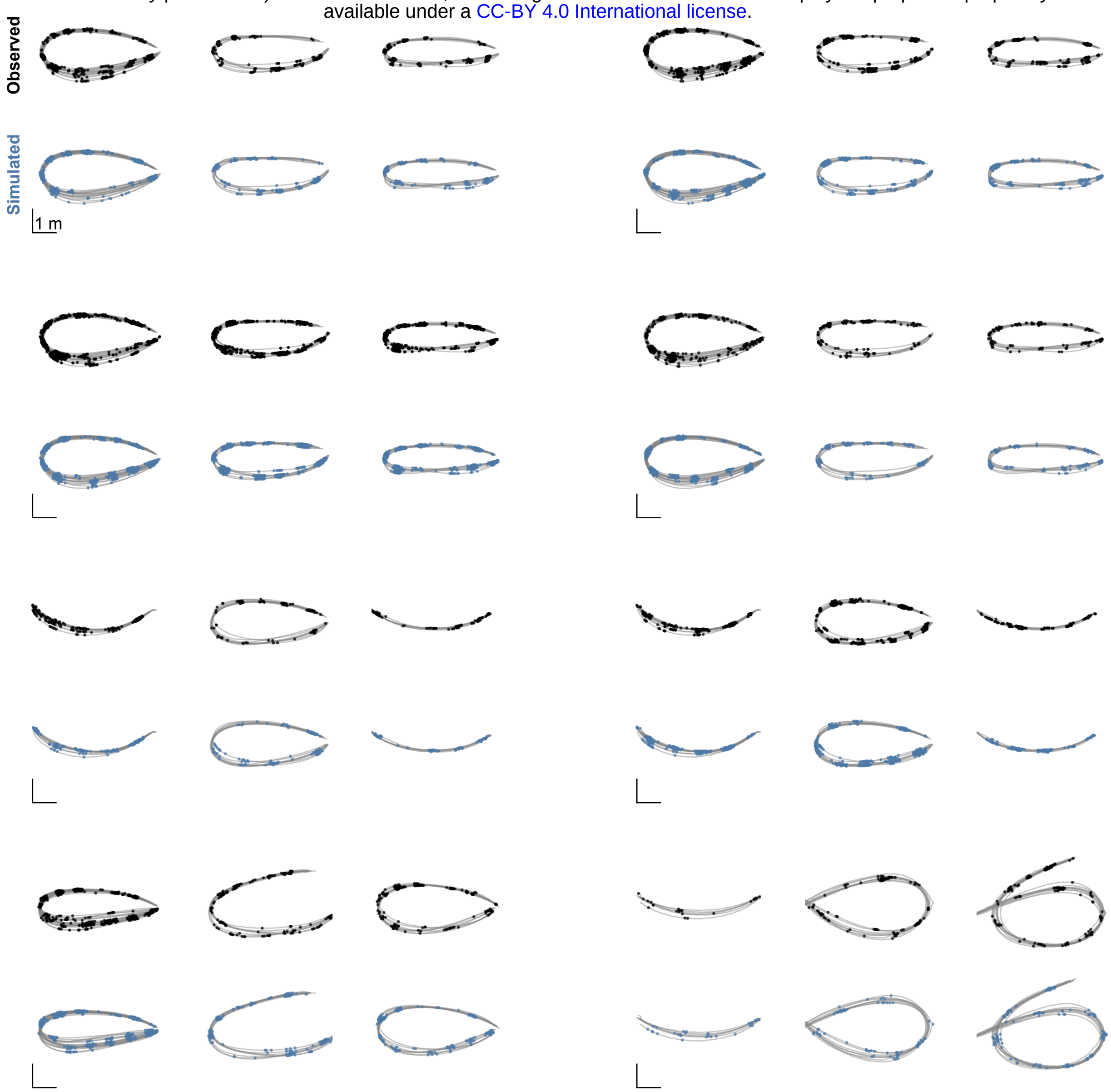

Figure S8. Additional examples of grid fitting on the plane of motion
Hexagonal grid fitting results for eight example grid cells on three structured flight paths each. Black dots indicate observed spike positions overlaid on flight trajectories (grey). Blue dots indicate simulated spike positions from the best-fit two-dimensional hexagonal grid defined on the plane of motion.

bioRxiv preprint doi: https://doi.org/10.64898/2026.06.10.728358; this version posted June 11, 2026. The copyright holder for this preprint (which was not certified by peer review) is the author/funder, who has granted bioRxiv a license to display the preprint in perpetuity. It is made available under a CC-BY 4.0 International license.

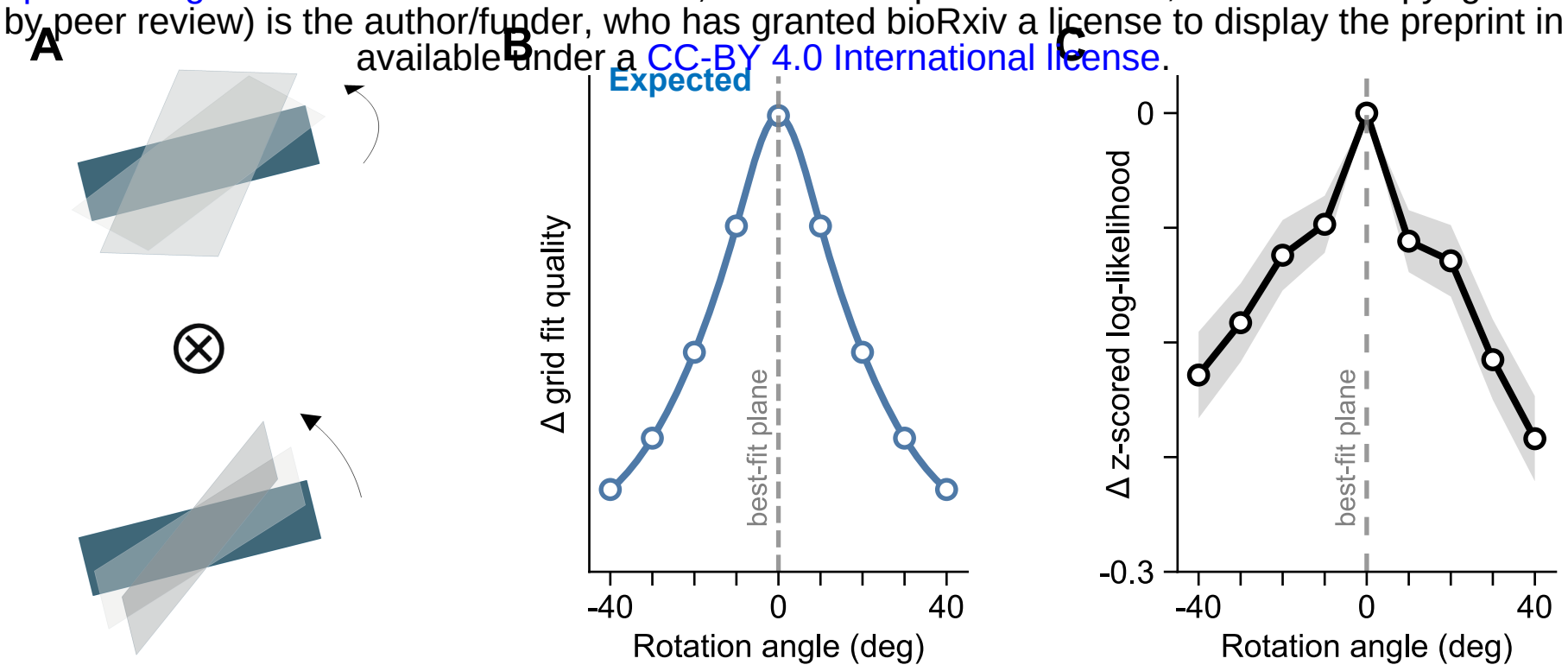

Figure S9. Grid fit quality decreases on planes rotated away from the plane of motion

(A) Schematic of candidate planes generated by rotating the best-fit plane of motion in azimuth and pitch, as shown in Figure 3K. **(B)** Expected decrease in grid fit quality on rotated planes if grid-cell representations are specifically aligned to the plane of motion, as shown in Figure 3L. **(C)** Observed change in z-scored log-likelihood across rotated planes, as in Figure 3M, for grid cells with significant Bonferroni-corrected grid fits on at least one of the 49 planes (Methods). This inclusion criterion avoids selection bias from defining significant grid cells only on the best-fit plane.

bioRxiv preprint doi: https://doi.org/10.64898/2026.06.10.728358; this version posted June 11, 2026. The copyright holder for this preprint (which was not certified by peer review) is the author/funder, who has granted bioRxiv a license to display the preprint in perpetuity. It is made available under a CC-BY 4.0 International license.

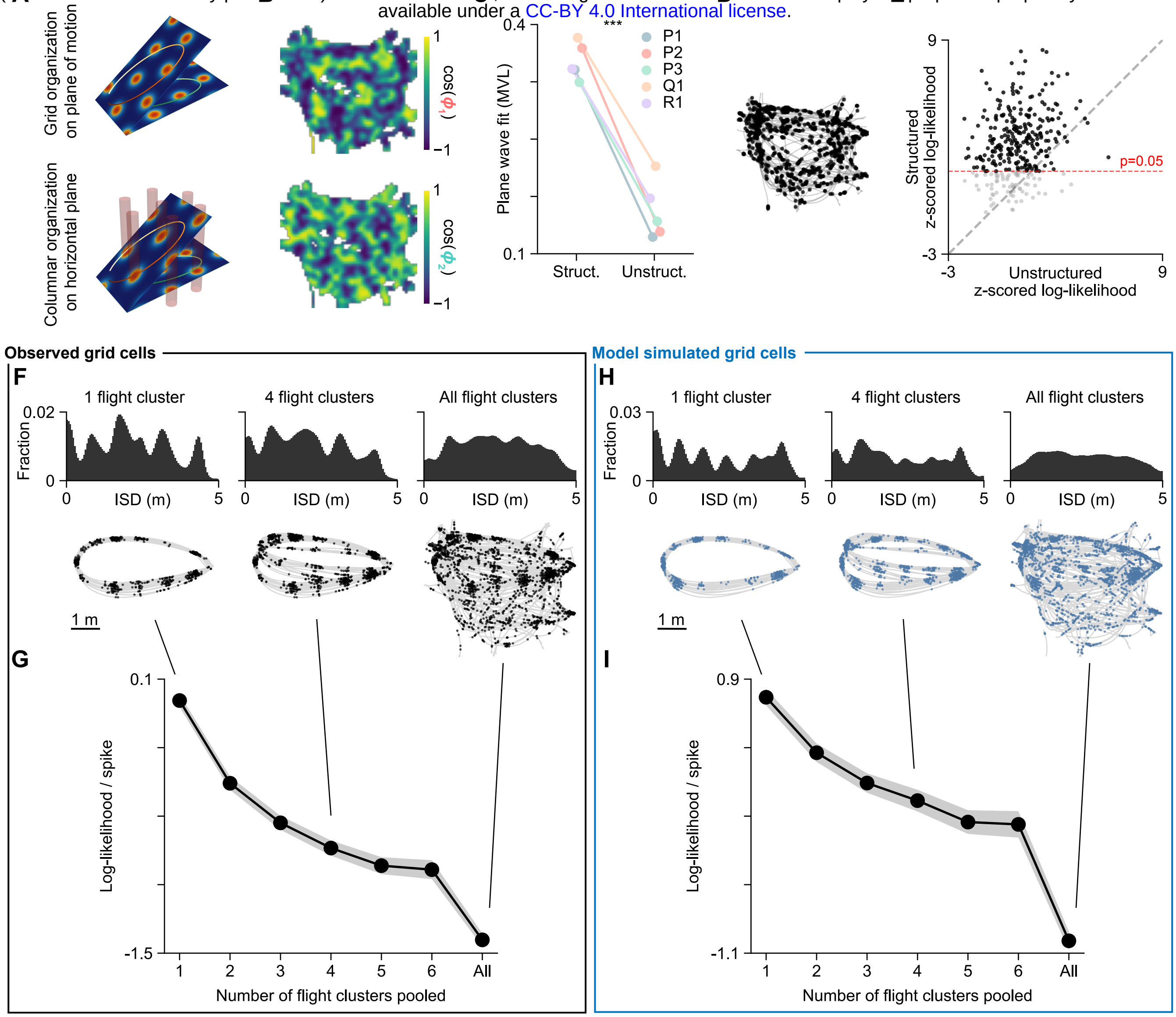

Figure S10. Reduced grid cell periodicity when pooling flights across different planes of motion **(A)** Schematic of grid-cell representations defined on the plane of motion of each flight path (top) versus a columnar organization relative to the horizontal plane (bottom). On structured flights confined to a single plane, both models make similar predictions. By contrast, during unstructured flights spanning multiple planes of motion, only the columnar model predicts clear grid structure when projected onto the horizontal plane. **(B)** Toroidal phase maps of ϕ_1 (top) and ϕ_2 (bottom) during unstructured flights. **(C)** Plane-wave fit mean vector length (MVL) for structured (left) and unstructured (right) flights across modules. Lines connect paired comparisons. $^{***}P < 0.001$. **(D)** Spikes (black) overlaid on pooled unstructured flight trajectories (grey). Note the absence of clear periodic structure when pooling across flights of different planes. **(E)** Paired scatter plot of grid-fit z-scored log-likelihood for grid-cell responses on structured flight paths versus unstructured flights. Red line, 95th percentile significance threshold on structured flight paths. Dashed grey line, unity line. **(F)** Example recorded grid-cell response obtained by pooling one (left), four (middle), or all (right) flight clusters. Top, inter-spike-distance (ISD) distribution for all spike pairs across the included flight clusters. Note the reduction in clear ISD distribution peaks as more flight clusters are combined. Bottom, spikes (black) over flight trajectories (grey). **(G)** Mean grid-fit log-likelihood per spike as a function of the number of pooled flight clusters for recorded grid cells (Methods). Grey shaded region denotes s.e.m. **(H)** Example simulated flight-plane-specific grid-cell response for one (left), four (middle), or all (right) flight clusters. Simulated grid cells were defined on the plane of motion of each flight trajectory (Methods). Top, ISD distribution as in f. Bottom, simulated grid cell spikes (blue) over flight trajectories (grey).

bioRxiv preprint doi: https://doi.org/10.64898/2026.06.10.728358; this version posted June 11, 2026. The copyright holder for this preprint (which was not certified by peer review) is the author/funder, who has granted bioRxiv a license to display the preprint in perpetuity. It is made available under a CC-BY 4.0 International license.

(I) Mean grid-fit log-likelihood per spike as a function of the number of pooled flight clusters for simulated flight-plane-specific grid cells. Grey shaded region denotes s.e.m..

bioRxiv preprint doi: https://doi.org/10.64898/2026.06.10.728358; this version posted June 11, 2026. The copyright holder for this preprint (which was not certified by peer review) is the author/funder, who has granted bioRxiv a license to display the preprint in perpetuity. It is made available under a CC-BY 4.0 International license.

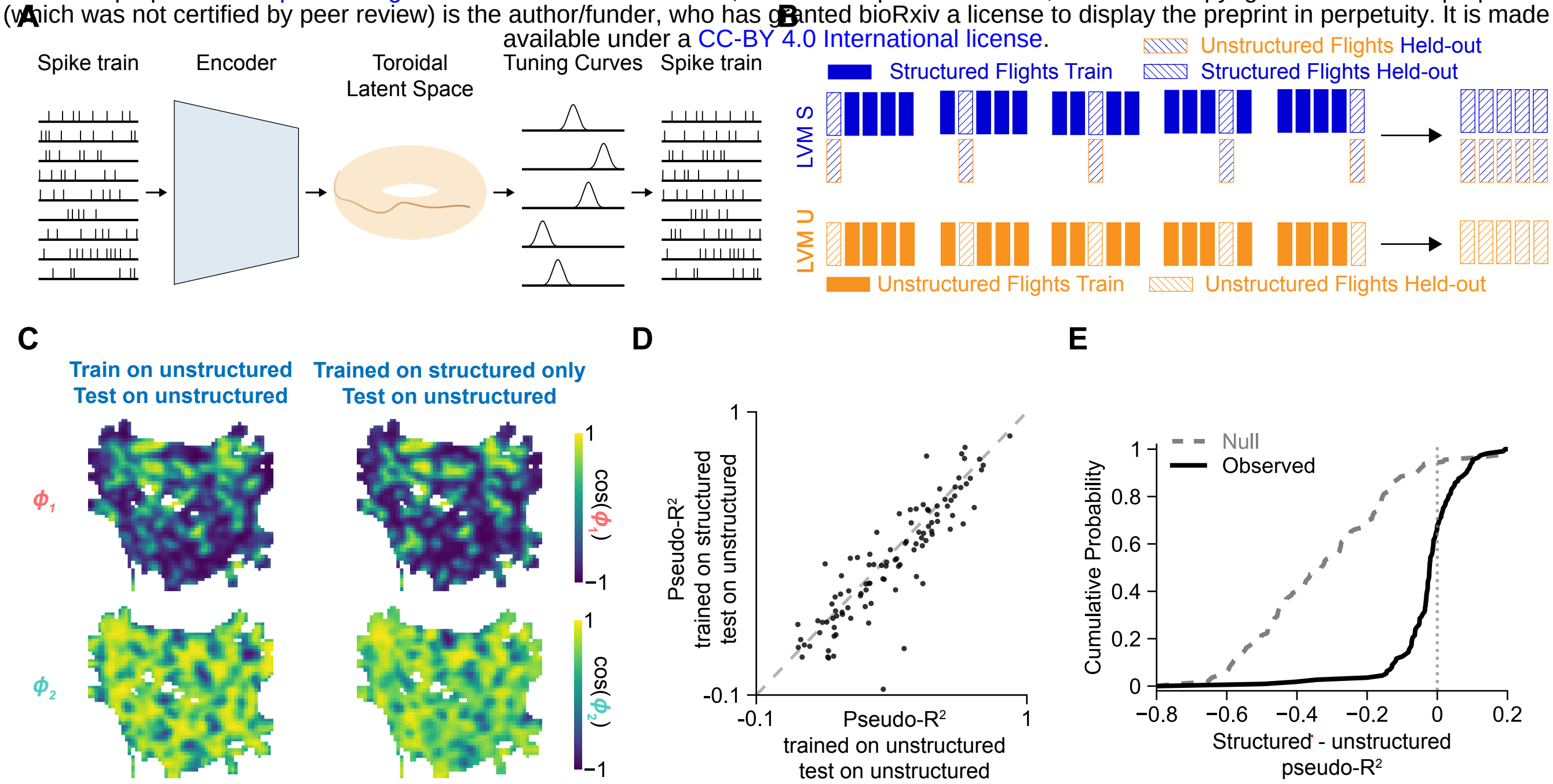

Figure S11. Toroidal latent dynamics generalize from structured to unstructured flights
(A) Schematic of the latent variable model (LVM) with toroidal latent space, based on the latent variable model by Bjerke et al.[57] (Methods). **(B)** Illustration of the 5-fold train-test procedure used to compare transfer performance of an LVM trained on structured flights only (blue, LVM S) versus a reference trained directly on unstructured flights (orange, LVM U). For each fold, structured and unstructured flights were split into training data, shown as solid bars, and held-out data, shown as striped bars (Methods). **(C)** Example toroidal phase maps of ϕ_1 (top) and ϕ_2 (bottom) during unstructured behaviors as predicted by LVM trained on all unstructured flights (left) and structured flights only (right). **(D)** Paired scatter plot of per-cell pseudo-R^2 scores on held-out unstructured flights for the LVM trained on structured flights only (y-axis) versus LVM trained on unstructured flights. Only cells with positive pseudo-R^2, indicating fit above the baseline null model, are shown (104 / 109 cells across modules shown). Dashed grey line, unity. **(E)** Cumulative distribution of pseudo-R^2 differences on held-out unstructured flights between the LVM trained on structured flights only and the LVM trained on unstructured flights. Dashed grey line indicates the cumulative distribution of the transfer performance of a constant firing-rate null model.

bioRxiv preprint doi: https://doi.org/10.64898/2026.06.10.728358; this version posted June 11, 2026. The copyright holder for this preprint (which was not certified by peer review) is the author/funder, who has granted bioRxiv a license to display the preprint in perpetuity. It is made available under a CC-BY 4.0 International license.

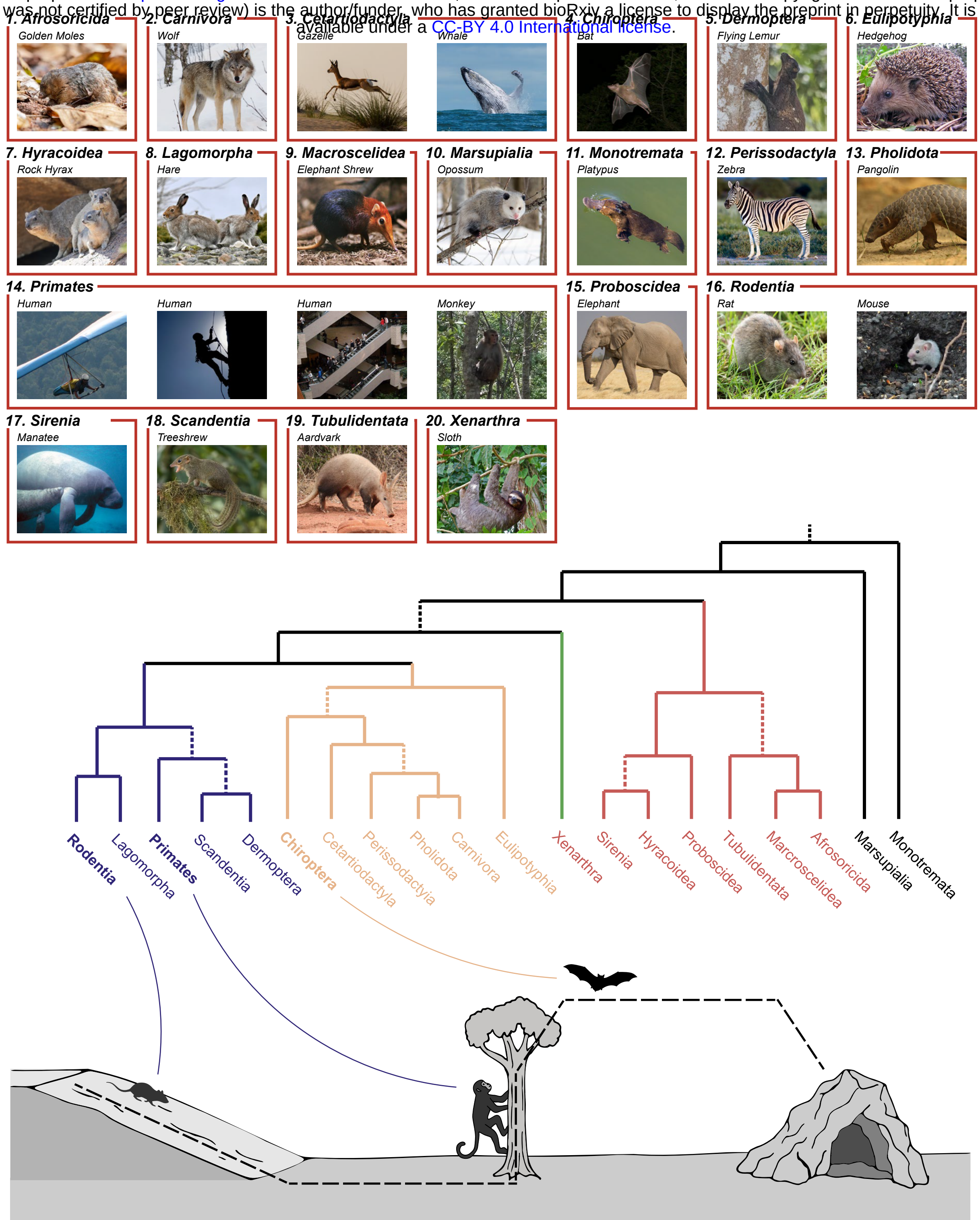

Figure S12. Behaviorally relevant movement subspaces in three-dimensional environments across mammals

Top, example species from 20 major mammalian lineages, illustrating the diversity of mammalian ecological niches and locomotor strategies. Middle, Evolutionary tree of these lineages, modified and adapted from Springer et al.[58]. Bottom, schematic examples illustrating how movement through three-dimensional environments can often be organized along locally planar subspaces defined by species-specific ecology, body plan, and mode of locomotion, including terrestrial travel along ground surfaces, arboreal movement along trunks and branches, and aerial flight through structured planes of motion. These examples illustrate that, although mammals inhabit three-dimensional worlds, behavior and environmental geometry often structure movement along lower-dimensional movement manifolds.
